# *Ixodes scapularis* and *Ixodes ricinus* tick cell lines respond to infection with tick-borne encephalitis virus: transcriptomic and proteomic analysis

**DOI:** 10.1186/s13071-015-1210-x

**Published:** 2015-11-18

**Authors:** Sabine Weisheit, Margarita Villar, Hana Tykalová, Marina Popara, Julia Loecherbach, Mick Watson, Daniel Růžek, Libor Grubhoffer, José de la Fuente, John K. Fazakerley, Lesley Bell-Sakyi

**Affiliations:** The Roslin Institute and Royal (Dick) School of Veterinary Studies, University of Edinburgh, Easter Bush, Midlothian, Scotland EH25 9RG UK; The Pirbright Institute, Ash Road, Pirbright, Surrey GU24 0NF UK; SaBio. Instituto de Investigación en Recursos Cinegéticos IREC-CSIC-UCLM-JCCM, Ronda de Toledo s/n, Ciudad Real, 13005 Spain; Faculty of Science, University of South Bohemia and Biology Centre, Institute of Parasitology, Czech Academy of Sciences, Branisovska 31, České Budějovice (Budweis), 37005 Czech Republic; Veterinary Research Institute, Hudcova 70, Brno, 62100 Czech Republic; Department of Veterinary Pathobiology, Center for Veterinary Health Sciences, Oklahoma State University, Stillwater, OK 74078 USA; Institute for Cancer Research, The Norwegian Radium Hospital, Oslo University Hospital, Oslo, 0377 Norway

**Keywords:** Tick, *Ixodes*, Flavivirus, Tick-borne encephalitis virus, Tick cell line, Innate immunity, Antiviral response

## Abstract

**Background:**

Ixodid ticks are important vectors of a wide variety of viral, bacterial and protozoan pathogens of medical and veterinary importance. Although several studies have elucidated tick responses to bacteria, little is known about the tick response to viruses. To gain insight into the response of tick cells to flavivirus infection, the transcriptomes and proteomes of two *Ixodes* spp cell lines infected with the flavivirus tick-borne encephalitis virus (TBEV) were analysed.

**Methods:**

RNA and proteins were isolated from the *Ixodes scapularis*-derived cell line IDE8 and the *Ixodes ricinus*-derived cell line IRE/CTVM19, mock-infected or infected with TBEV, on day 2 post-infection (p.i.) when virus production was increasing, and on day 6 p.i. when virus production was decreasing. RNA-Seq and mass spectrometric technologies were used to identify changes in abundance of, respectively, transcripts and proteins. Functional analyses were conducted on selected transcripts using RNA interference (RNAi) for gene knockdown in tick cells infected with the closely-related but less pathogenic flavivirus Langat virus (LGTV).

**Results:**

Differential expression analysis using DESeq resulted in totals of 43 and 83 statistically significantly differentially-expressed transcripts in IDE8 and IRE/CTVM19 cells, respectively. Mass spectrometry detected 76 and 129 statistically significantly differentially-represented proteins in IDE8 and IRE/CTVM19 cells, respectively. Differentially-expressed transcripts and differentially-represented proteins included some that may be involved in innate immune and cell stress responses. Knockdown of the heat-shock proteins HSP90, HSP70 and gp96, the complement-associated protein Factor H and the protease trypsin resulted in increased LGTV replication and production in at least one tick cell line, indicating a possible antiviral role for these proteins. Knockdown of RNAi-associated proteins Argonaute and Dicer, which were included as positive controls, also resulted in increased LGTV replication and production in both cell lines, confirming their role in the antiviral RNAi pathway.

**Conclusions:**

This systems biology approach identified several molecules that may be involved in the tick cell innate immune response against flaviviruses and highlighted that ticks, in common with other invertebrate species, have other antiviral responses in addition to RNAi.

**Electronic supplementary material:**

The online version of this article (doi:10.1186/s13071-015-1210-x) contains supplementary material, which is available to authorized users.

## Background

Ticks are hematophagous ectoparasites that are second only to mosquitoes in their importance as vectors of viral, bacterial and protozoan pathogens that cause human disease, and they are probably the most important vectors of livestock disease worldwide [1].

Tick-borne encephalitis virus (TBEV), a member of the family *Flaviviridae*, is a medically-important virus transmitted by ticks. TBEV is one of the most important tick-borne viruses in Europe, Russia and many parts of Asia, causing tick-borne encephalitis in humans with an estimated annual number of disease cases >10,000 [2, 3]. The Western European subtype of TBEV is transmitted by *Ixodes ricinus* ticks in Central, Eastern and Northern Europe, whereas the Siberian and Far-Eastern subtypes are transmitted by *Ixodes persulcatus* ticks in Siberia, parts of Russia, Latvia and Finland and the latter subtype additionally in Central and Eastern Asia including China and Japan [4, 5]. Other tick species may also transmit TBEV under certain ecological conditions [5]; however it is not known if *Ixodes scapularis* ticks found in the United States, where TBEV does not occur, are capable of transmitting the virus.

Langat virus (LGTV), a close relative of TBEV, was isolated from *Ixodes granulatus* ticks in Malaysia [6]. Although the virus is antigenically closely related to TBEV, there are no reports of naturally-acquired cases of human disease caused by LGTV. The attenuated LGTV strain E5 was tested as a candidate live vaccine against TBEV in animals and human volunteers. It resulted in high levels of neutralising antibodies which cross-reacted with TBEV, Powassan virus and Kyasanur Forest disease virus [7, 8]. Due to its close antigenic relationship with TBEV, low pathogenicity and lack of naturally-occurring cases of disease in humans and animals, LGTV is a useful experimental model for more virulent tick-borne flavivirus infections.

Most knowledge of the response of arthropods to microorganisms has been obtained from studies in insects. These have revealed the involvement in the antiviral response of several signaling pathways including RNA interference (RNAi) [9, 10], Toll, Immune deficiency (IMD), and Janus kinase-signal transducers and activators of transcription (JAK/STAT), as well as melanisation, autophagy and possibly heat shock proteins (HSPs) (reviewed by [11–14]). RNAi, Toll, IMD and JAK/STAT pathway components have been identified in the genome of the tick *I. scapularis* [15, 16], but in comparison to insects there is only limited knowledge on tick innate immune responses to pathogen infection [15, 17–19]. A recent study reported a role for the JAK/STAT pathway in *I. scapularis* ticks during *Anaplasma phagocytophilum* infection [20]. This study showed that silencing of STAT or JAK, but not Toll-1, TAK1 or TAB1, which are components of the Toll and IMD pathways, resulted in an increase in *A. phagocytophilum* in infected ticks and that the JAK/STAT pathway controls bacterial infection by regulating the expression of antimicrobial peptides of the 5.3 kD gene family. Other important regulatory molecules with a possible role in tick innate immune responses include RNA-dependent RNA polymerase, subolesin and ubiquitin-related molecules [21–24].

The only antiviral innate immune response described to date in ticks is RNAi [25, 26]. RNAi has been efficiently used for gene knockdown in ticks and tick cell lines [27–29]. Tick cell lines have been used as tools to understand LGTV and TBEV interactions with their vectors [30–38]. Recently, Dicer (Dcr) and several orthologues of Argonaute (Ago) 2, a key member of the exogenous siRNA pathway in insects, were identified in ticks and Dcr 90, Ago 16 and Ago 30 were shown to mediate an antiviral response [38].

The present study was carried out with the aim of identifying transcripts and proteins with a possible role in tick innate antiviral responses. We first characterised TBEV infection in the tick cell lines IDE8 derived from the only tick species with a sequenced genome, *I. scapularis*, and IRE/CTVM19 derived from *I. ricinus*, a natural vector of TBEV. We then investigated differences in transcript and protein abundance between TBEV-infected and mock-infected tick cells using the Illumina HiSeq2000 platform and LC-MS/MS, respectively. Statistically significantly differentially-expressed transcripts and differentially-represented proteins were identified, annotated and grouped according to their biological function. Finally, using LGTV which could be handled at a lower level of biosafety containment than TBEV, we silenced selected transcripts and proteins by RNAi, to elucidate their effect on virus replication and production.

## Methods

### Ethics statement

This study was carried out in strict accordance with the Czech national law and guidelines on the use of experimental animals and protection of animals against cruelty (the Animal Welfare Act Number 246/1992 Coll.). The protocol was approved by the Committee on the Ethics of Animal Experiments of the Institute of Parasitology and of the Departmental Expert Committee for the Approval of Projects of Experiments on Animals of the Czech Academy of Sciences (Permit Number: 165/2010).

### Tick and mammalian cell lines

The *I. scapularis-*derived cell line IDE8 [39] was maintained in ambient air at 32 °C in L-15B medium [40] supplemented with 10 % tryptose phosphate broth (TPB), 5 % fetal calf serum (FCS), 0.1 % bovine lipoprotein (MP Biomedicals), 2 mM L-glutamine and 100 units/ml penicillin and 100 μg/ml streptomycin (pen/strep). The *I. ricinus*-derived cell line IRE/CTVM19 [41] was maintained in ambient air at 28 °C in L-15 (Leibovitz) medium supplemented with 10 % TPB, 20 % FCS, 2 mM L-glutamine and pen/strep [42]. Baby hamster kidney (BHK-21) cells (C-13, ATCC, cat: CCL-10) and African green monkey kidney epithelial (Vero) cells (ECACC, cat: 84113001) were grown at 37 °C in a humidified atmosphere of 5 % CO_2_ in air. Porcine kidney stable (PS) cells were grown at 37 °C in ambient air [43]. BHK-21 cells were maintained in Glasgow Minimal Essential Medium (GMEM) supplemented with 5 % newborn calf serum (NBCS), 10 % TPB, 2 mM L-glutamine and pen/strep (GMEM/5 % NBCS). Vero cells were grown in Dulbecco’s Minimal Essential Medium containing 10 % FCS and pen/strep. PS cells were maintained in L-15 (Leibovitz) medium supplemented with 3 % NBCS, 2 mM L-glutamine, pen/strep and 0.25 μg/ml amphotericin B (L-15/3 % NBCS).

### Virus strains, propagation and virus titration

The TBEV strain Neudoerfl was kindly provided by Professor F.X. Heinz, Institute of Virology, Medical University of Vienna, Austria, and had been passaged five times by intracranial infection of suckling mice before use in the present study. Suckling CD1 mice were intracranially infected with 1 μl of TBEV-infected mouse brain suspension corresponding to 100 plaque-forming units (PFU) per mouse, or mock-infected with the same volume of uninfected mouse brain suspension. After the onset of symptoms, 4 to 5 days post infection (p.i.), the TBEV-infected mice were euthanised and the brains removed. The mock-infected mice were euthanised 2 days later, to prevent any possibility of cross contamination while handling the samples. The brains were homogenised in L-15/3 % NBCS to obtain a 20 % mouse brain suspension (w/v) using a Tissue Lyser II (Retsch) at 30 Hz (30/s) for 2 min. The homogenate was then centrifuged for 10 min at 16,000 x g at 4 °C and the clarified supernatant was used for infection of tick cell lines.

The LGTV strain TP-21 was kindly provided by Dr Sonja Best, Laboratory of Virology, Rocky Mountain Laboratories, NIAID, NIH, Hamilton, Montana, USA and was propagated in Vero cells prior to being used for infection. TBEV was titrated on PS cells as described previously [44, 45]; the titre of the stock used in the experiments was 6x10^7^ PFU/ml. LGTV was titrated on BHK-21 cells in 12-well plates using Avicel (RC-581, FMC Biopolymer) as an overlay. In brief, cells were seeded at a density of 1.5x10^5^ cells per well in GMEM/5 % NBCS and incubated overnight. When the cells were 80 % confluent, medium was removed and replaced with supernatant of test samples which had been 10-fold serially diluted in GMEM/2 % NBCS. After incubation on a shaker for 1 h, cells were overlaid with 1 ml of Avicel suspension (1.2 g Avicel in 100 ml PBS) mixed in a 1:1 ratio with 2x Minimal Essential Medium (Gibco) supplemented with 5 % FCS. Cells were incubated for 4 days, fixed in 10 % neutral buffered formaldehyde (Leica), stained with 0.1 % aqueous toluidine blue for 30 min and plaques were counted. The titre of the LGTV stock used in the experiments was 2x10^6^ PFU/ml.

### Infection of tick cell lines

For establishing a TBEV growth curve, both tick cell lines were seeded at a density of 5x10^5^ cells per ml in 2 ml total medium volume in flat-sided tubes (Nunc) and 24 h later were infected with TBEV diluted in the respective tick cell growth medium to a multiplicity of infection (MOI) of 5. After infection, supernatant was collected over a 10 day time-period for virus titration. Tick cells used for transcriptomic and proteomic analysis were seeded at a density of 1.5x10^6^ (IDE8) or 1x10^6^ (IRE/CTVM19) cells per ml in flat-sided tubes and were infected 24 h later with TBEV at MOI 5. Preliminary experiments (data not shown) revealed that RNA and protein yields from equivalent numbers of cells were higher from IRE/CTVM19 cells than from IDE8 cells, presumably because IRE/CTVM19 cells are larger than IDE8 cells (authors’ observations), and established that the cell densities used were the minimum required to produce acceptable RNA and protein yields. After incubation for the required time cells were harvested by pipetting for analysis as indicated below. To validate differential expression of transcripts in IDE8 and IRE/CTVM19 cells upon LGTV infection, cells were seeded at a density of 5x10^5^ per ml in flat-sided tubes, mock-infected or infected 24 h later with LGTV at MOI 5 and the medium was changed 2 h p.i.. At 2 and 6 days p.i., cells were harvested and RNA was isolated for qRT-PCR analysis as described below.

### Immunostaining

Tick cells were seeded at a density of 5x10^5^ cells per ml on 12 mm diameter glass coverslips in 24-well plates, incubated overnight and two replicate wells were infected with TBEV at each of MOI 0.1, 1 and 5. The immunostaining procedure was carried out in the 24-well plates. At day 2 p.i. the cells on the coverslips were washed in PBS, fixed in 10 % neutral buffered formaldehyde for 1 h and washed for 5 min in PBS. The cells were then permeabilised with 300 μl of 0.3 % TritonX-100 for 30 min and subsequently with 0.1 % SDS for 10 min. After permeabilisation, the cells were washed in PBS and then blocked with 300 μl of 1 % bovine serum albumin (BSA) in PBS for 60 min. The blocking solution was removed and the primary antibody, Anti-Flavivirus Group antigen antibody (clone D1-4G2-4-15, Millipore, recognising the E protein of flaviviruses) diluted 1:100 in 1 % BSA, was added and incubated for 2 h at room temperature. The cells were washed three times for 5–10 min in PBS and the secondary antibody, Goat anti-Mouse IgG (H + L) DyLight 488 conjugate (Pierce Thermo Scientific) diluted 1:1000 in 1 % BSA, was added and incubated for 1 h. After three washes of 5 min each in PBS, the coverslips were mounted on glass microscope slides with Vectashield HardSet mounting medium containing DAPI (Vector Laboratories). Images were taken of randomly-selected fields using an Olympus Fluoview FV10 confocal microscope and the percentage of green cells determined by visual counting of DyLight 488-positive and negative cells.

### RNA and protein isolation

IDE8 and IRE/CTVM19 cells were seeded at densities of, respectively, 1x10^6^ and 1.5x10^6^ cells per ml into 24 tubes per cell line and, 24 h later, 12 tubes per cell line were infected with TBEV at MOI 5 and 12 tubes were mock-infected with the same volume (300 μl) of diluted uninfected mouse brain suspension. On each of days 2 and 6 p.i., cells from six tubes per treatment were harvested by pipetting and the cell suspension from each replicate tube was split into two aliquots of 1 ml, which were both centrifuged at 500 x g for 5 min and the supernatants discarded. One aliquot from each replicate tube was used for RNA isolation using 1 ml of TriReagent (Sigma) according to the manufacturer’s instructions. To improve RNA purity, RNA samples were further purified using the RNeasy Mini kit (Qiagen) according to the manufacturer’s instructions. RNA samples were stored at −80 °C. The second aliquot from each replicate tube was used for protein isolation as follows. The cell pellet was washed twice with ice-cold PBS and resuspended in 350 μl ice-cold PBS supplemented with 1 % Triton X-100, 50 μl cOmplete, Mini, EDTA-free Protease Inhibitor Cocktail (Roche) and 3.5 μl Halt Phosphatase Inhibitor Cocktail (Thermo Scientific Pierce). After incubation for 1 h on ice, the cell suspension was homogenised at 4 °C using a micro pestle and centrifuged at 200 x g for 5 min to remove cell debris. Supernatants were collected and protein concentration was determined with the BCA protein assay kit (Thermo Scientific Pierce) using BSA as standard. Samples were stored at −80 °C until use.

Protein quality was tested by SDS-PAGE with subsequent Coomassie staining as follows. Protein samples were mixed 1:1 (v/v) with 2x Laemmli buffer (Biorad) supplemented with 5 % β-mercaptoethanol (Sigma), heated at 96 °C for 10 min and then loaded onto discontinuous SDS-PAGE (0.75 mm thick, 4 % stacking and 12 % resolving) gels. The gels were run at 40 V for 30 min followed by 120 V for 30 min. For staining gels, a solution consisting of 0.25 g Coomassie Brilliant Blue R-250 (Thermo Scientific Pierce) dissolved in 40 % water, 50 % methanol and 10 % glacial acetic acid was prepared. The gels containing protein were immersed in staining solution for 3 h prior to de-staining in a solution containing 50 % methanol, 40 % water and 10 % glacial acetic acid. The de-staining solution was changed several times until the protein bands were clearly visible. Samples showing good protein quality with clear, distinct bands and widely-distributed molecular masses were considered suitable for proteomic analysis.

### RNA sequencing and assembly

RNA integrity was assessed using the RNA 6000 Nano Kit (Agilent) according to the manufacturer’s instructions and tested for RNA integrity using a 2100 Bioanalyser (Agilent) according to the manufacturer’s instructions. Before sequencing, infection levels were measured by qRT-PCR using primers targeting the TBEV NS5 protein (Additional file 1). Aliquots of only those samples showing satisfactory RNA quality, and presence or absence of TBEV infection in the case of infected cells and mock-infected controls respectively, were pooled according to time-point, cell line and condition. The pooled RNA samples containing total RNA were processed by ARK-Genomics (http://www.ark-genomics.org/) according to the Truseq RNA sample guide 1500813 (Illumina Inc). In brief, mRNA molecules containing poly(A) tails were purified from total RNA using poly-T oligo‐attached magnetic beads. The resulting mRNA was fragmented, first and second strand cDNAs were synthesised, ends repaired and adapters ligated. After PCR amplification, the cDNA library was quantified, multiplexed and sequenced on the HiSeq2000 platform, generating paired end reads of approximately 2 x 100 bp in length. The reads were sorted into samples according to cell line, time-point and treatment using the software CASAVA 1.8 (Illumina, https://support.illumina.com/sequencing/sequencing_software/casava.ilmn). Reads obtained from the *I. scapularis*-derived cell line IDE8 were mapped with TopHat 2.0.3 [46] against the *I. scapularis* reference genome (iscapularis.SUPERCONTIGS-Wikel.IscaW1.fa). Counts of reads mapping to the genome were generated with HTSeq count 0.5.3p9 (http://www-huber.embl.de/users/anders/HTSeq/doc/count.html). The unmapped reads were *de novo* assembled with CLC genomic workbench 5.1 (http://www.clcbio.com/products/clc-genomics-workbench/) and mapped with BWA 0.6.1 [47] against the mapped, filtered (5x 400b) reads for generating counts using a Perl script. The reads obtained from the *I. ricinus* cell line IRE/CTVM19 were *de novo* assembled as described for the unmapped reads from IDE8. Only reads mapping unambiguously to contigs were counted.

### Differential gene expression analysis and annotation

Each assembled contig was assumed to represent a transcript and, since the majority of reads generated during sequencing mapped unambiguously, it was assumed that the count data reflected the expression of each transcript. As reported in previous studies [48–51], we did not use biological replicates for RNA-seq but used pooled RNA isolated from replicate samples; the algorithm used to quantitate transcriptomics data allows the use of non-replicated samples [52, 53]. Differential gene expression was analysed using DESeq in R following the script for working without replicates [52]. DESeq uses a very conservative approach in calling statistical significance in samples without biological replicates. This results in fewer transcripts being called statistically significant; thus some important transcripts might have been missed, whereas the transcripts that were included were strongly supported. Transcripts that were greater than log_2_ 2-fold differentially expressed, and those statistically significantly differentially expressed, were annotated first using Blast2GO [54] with a Blastx algorithm against the NCBI nr database using a threshold of E-value < 10^−6^ as cut-off. Those sequences which did not result in any blast hits with Blast2GO were blasted manually using Blastx and Blastn algorithms against the nr and nt NCBI databases and were included when they showed more than 50 % coverage and more than 25 % sequence similarity. All sequences obtained by either of the two approaches were additionally blasted against the UniProt/Swiss-Prot and VectorBase databases to retrieve ontology information, including ontology information for conserved domains provided by NCBI and UniProt. For the statistically significantly differentially-expressed transcripts, literature research was performed in addition to database information retrieval to assign biological process groups.

### Proteomic analysis

For those samples which passed both the RNA and protein quality checks in each experimental group, protein extracts equivalent to 100 μg for each group, obtained by pooling equal aliquots from the replicates, were suspended in 100 μl of Laemmli buffer supplemented with 5 % β-mercaptoethanol and applied to 1.2 cm-wide wells of a conventional discontinuous SDS-PAGE gel (0.75 mm thick, 4 % stacking, and 12 % resolving). The electrophoretic run was stopped as soon as the dye front entered 3 mm into the resolving gel. The whole proteome, concentrated within the stacking/resolving gel interface, was visualised using Bio-Safe Coomassie Stain G-250 (BioRad), excised and cut into cubes of 2 x 2 mm. The gel pieces were destained in a 1:1 mixture of acetonitrile and water and digested overnight at 37 °C with 60 ng/ml sequencing grade trypsin (Promega, Madison, WI) as described previously [55]. Trifluoroacetic acid was added to a final concentration of 1 % to stop digestion, and peptides were desalted onto OMIX Pipette tips C18 (Agilent Technologies, Santa Clara, CA, USA) as described previously [56], dried down and stored at −20 °C until required for mass spectrometry analysis. The desalted protein digests were resuspended in 0.1 % formic acid and analysed by reversed phase liquid chromatography coupled to mass spectrometry (RP-LC-MS/MS) using an Easy-nLC II system coupled to an ion trap LTQ-Orbitrap-Velos-Pro mass spectrometer (Thermo Scientific, San Jose, CA, USA). The peptides were concentrated (on-line) by reverse phase chromatography using a 0.1 mm × 20 mm C18 RP precolumn (Thermo Scientific), and separated using a 0.075 mm x 100 mm C18 RP column (Thermo Scientific) operating at 0.3 μl/min. Peptides were eluted using a 180-min gradient from 5 % to 40 % solvent B in solvent A (Solvent A: 0.1 % formic acid in water, solvent B: 0.1 % formic acid, 80 % acetonitrile in water). ESI ionisation was carried out using a nano-bore emitters stainless steel ID 30 μm (Thermo Scientific) interface. Peptides were detected in survey scans from 400 to 1600 atomic mass units (amu, 1 μscan), followed by fifteen data-dependent MS/MS scans (Top 15), using an isolation width of 2 mass-to-charge ratio units, normalised collision energy of 35 %, and dynamic exclusion applied during 30 s periods.

### Proteomic data analysis and annotation

Peptide identification from the MS/MS raw data was carried out using the SEQUEST algorithm (Proteome Discoverer 1.3, Thermo Scientific). Database searches were performed against UniProt-Arthropoda.fasta and UniProt-Flaviviridae.fasta. The following constraints were used for the searches: tryptic cleavage after Arg and Lys, up to two missed cleavage sites, and tolerances of 10 ppm for precursor ions and 0.8 Da for MS/MS fragment ions, and the searches were performed allowing optional methionine oxidation and cysteine carbamidomethylation. Searches were performed against a decoy database in an integrated decoy approach. A false discovery rate (FDR) < 0.01 was considered as a condition for successful peptide assignments and at least 2 peptides per protein in at least one of the samples analysed was the condition for subsequent protein identification (Additional file 2). The total number of peptide assignments for each protein were normalised against the total number of peptide assignments in each sample (control and infected tick cell lines at days 2 and 6 p.i.) and differential representation of individual proteins between different samples was determined using Chi-square test statistics with Bonferroni correction in the IDEG6 software (http://telethon.bio.unipd.it/bioinfo/IDEG6 form/) (*p* < 0.05) as published [56]. Samples with a p-value equal to or lower than 0.05 were considered statistically significant. Statistically significantly differentially-represented proteins were allocated to biological process groups using ontology information available on the UniProt/Swiss-Prot and Panther databases, including information for conserved domains. Information was curated manually through literature search.

### Reverse transcription

For verification of infection status of TBEV-infected and mock-infected cells, 1 μg of total RNA was reverse-transcribed using Superscript III (Invitrogen) and random hexamers according to the manufacturer’s instructions. For verification of RNA-Seq data and knockdowns followed by LGTV infection, 1 μg of total RNA was reverse-transcribed using the High-Capacity cDNA Reverse Transcription kit (Applied Biosystems) according to the manufacturer’s instructions.

### Verification of TBEV infection and RNA-Seq data by qRT-PCR

TBEV RNA levels were measured by qRT-PCR with TBEV NS5-specific primers (Additional file 1) using FastStart SYBR Green Master Mix (Roche) according to the manufacturer’s instructions with a final reaction volume of 20 μl and a temperature profile of 95 °C for 5 min, 95 °C for 20s, 55 °C for 20s, 72 °C for 15 s and 95 °C for 20s. For calculating the TBEV infection levels of transcriptomic samples, TBEV NS5 copy numbers were calculated using a linearised plasmid encoding the TBEV NS5 protein as standard in a standard curve method as follows. The linearised plasmid was 9 x 10-fold serially diluted starting with 2 ng and the corresponding copy numbers were entered into the Rotor-GENE software which generated the standard curve and calculated the copy numbers for each unknown sample automatically. The number of copies of the linearised plasmid was calculated using the following formula [57]:

$$ \mathrm{Number}\ \mathrm{of}\ \mathrm{copies} = \kern0.5em \frac{amount\kern0.5em  of\kern0.5em  plasmid\kern0.5em \left(n\mathit{\mathsf{g}}\right)\kern0.5em \times \kern0.5em 6.022\kern0.5em x\kern0.5em 1{0}^{23}\kern0.5em \left(\frac{ mol ecules}{mol}\right)}{length\kern0.5em (bp)\kern0.5em \times \kern0.5em 1\kern0.5em x\kern0.5em {10}^9\kern0.5em \left(\frac{n\mathit{\mathsf{g}}}{\mathit{\mathsf{g}}}\right)\kern0.5em \times \kern0.5em 660\kern0.5em \left(\frac{\mathit{\mathsf{g}}}{mol\kern0.5em  of\kern0.5em  bp}\right)} $$

The gene coding for TBEV NS5 protein was cloned into the pJET vector using the CloneJET PCR Cloning Kit (Fermentas) according to the manufacturer’s instructions. In brief, the plasmid pTND/ΔME [58] was linearised, purified and used as DNA template for amplification of TBEV NS5 using KOD polymerase (Novagen). An aliquot of the 187 bp PCR product was visualised by gel electrophoresis and, since only one band with the correct size was visible, the non-purified product was directly used for ligation. For ligation, 10 μl 2x reaction buffer, 2 μl non-purified PCR product, 1 μl pJET1.2 blunt cloning vector, 6 μl nuclease-free water and 1 μl T4 DNA ligase were mixed by vortexing. The ligation mixture was incubated for 5 min at room temperature before using directly for transformation of DH5α. To check if the correct insert was cloned into the vector, the plasmid was linearised and sent for sequencing to GATC Biotech (London, UK).

For verification of RNA-Seq data, primers for 12 transcripts (Additional file 1) were designed, using as template species-specific sequences or identical regions from sequences common to both *I. scapularis* and *I. ricinus* obtained by HiSeq2000. The same samples from which aliquots had been pooled for the transcriptome profiling were used individually for qRT-PCR analysis. Beta actin and ribosomal protein L13A were used as housekeeping genes for normalisation. Primer efficiencies were calculated for each primer and the quantity of gene transcripts in infected samples relative to controls was calculated using the 2^-∆∆CT^ method [59, 60].

### dsRNA production and silencing of tick transcripts

Long dsRNA transcripts (407 - 615 bp in length) specific to genes from each cell line were produced from PCR products flanked by T7 promoter sequences using the MegaScript RNAi kit (Ambion). In brief, cDNA generated by reverse transcription from total RNA of tick cells or from the plasmid pIRES2-eGFP (Clontech) was used as template to generate specific PCR products using T7 primers (Additional file 1) by PCR. PCR products were gel-purified and sequenced. The gel-purified PCR products were subjected to an additional PCR amplification and were then transcribed using the MegaScript kit according to the manufacturer’s instructions.

For knockdown experiments, LGTV was used at a low MOI to ensure that not all cells would be infected initially, thereby allowing virus to spread from cell to cell which might enhance detection of any effect of transcript knockdown on virus replication and/or production. Cell lines IDE8 and IRE/CTVM19 were seeded at a density of 5x10^5^ cells per ml in 24-well plates and were incubated in humidified self-sealing polythene bags. For IDE8 cells, 300 ng of dsRNA was added to the supernatant twice, at 8 h and 48 h post-seeding. Approximately 72 h post-seeding, cells were infected with LGTV at MOI 0.01; 48 h later supernatant was collected for plaque assay and cells were harvested for RNA extraction. To achieve a good knockdown in IRE/CTVM19 cells, cells were transfected 24 h post-seeding with 400 ng of dsRNA using Lipofectamine® 2000 (Invitrogen) as previously described [29] and, after incubation for a further 48 h, were infected with LGTV at MOI 0.5. At 24 h p.i supernatant was collected for plaque assay and RNA was extracted using TriReagent as above. Non-specific dsRNA encoding eGFP was used as a negative control, to provide a baseline level of activation above which the effect of the specific exogenous dsRNA was measured. Additional controls, in which samples were not treated with dsRNA prior to infection, were included to test whether or not addition of any non-specific dsRNA triggers an innate immune response in tick cells and to provide a baseline for virus replication and virus titres in untreated cells. For the detection of Ago and Dcr knockdowns, PCR was carried out (95 °C for 2 min, 95 °C for 30 s, primer set specific annealing temperature (Additional file 1) for 30s, 72 °C for 50 s, 72 °C for 7 min) using GoTaq reaction mix (Promega), according to the manufacturer’s instructions, together with 2 μl of the cDNA reaction and the corresponding primers (Additional file 1).

PCR products were run on a 1 % agarose gel and gel images were taken and used to quantify mRNA knockdown with Image Lab software (BioRad) normalised to beta actin. Gene knockdowns and LGTV RNA levels were measured by qRT-PCR with, respectively, target gene-specific primers or LGTV NS5-specific primers (Additional file 1), using FastStart Universal SYBR Green Master (Rox) (Roche) with a temperature profile of 95 °C for 10 min, 95 °C for 15 or 20s, specific annealing temperature for 20 or 30s, 72 °C for 15 or 30s and 95 °C for 15 s. All qRT-PCR reactions were followed by melting curve generation (60-95 °C) to confirm amplification specificity. Primer efficiencies were calculated for each primer and the quantity of gene transcripts in infected samples relative to controls was calculated using the 2^-∆∆CT^ method [59, 60].

### Statistical analysis of gene knockdown experiments

Gene knockdowns were done in quadruplicate in three independent experiments. Only those samples in which a knockdown occurred were included in subsequent statistical analysis. Analysis was done in GraphPad Prism (http://www.graphpad.com/scientific-software/prism/). Statistical significance of the three independent experiments was analysed using the two-way Analysis of Variance Fisher’s LSD test (*P =* 0.05).

## Results and discussion

### Characterisation of TBEV growth in tick cells

**Fig. 1:**
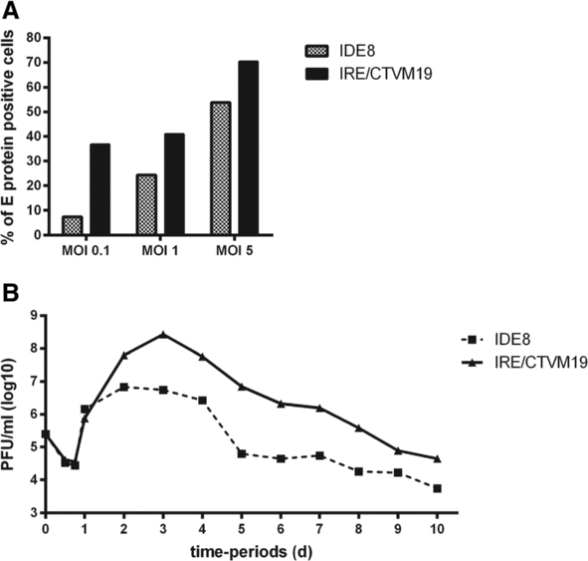
Characterisation of TBEV infection in IDE8 and IRE/CTVM19 cells. **a** Percentage of E protein-positive tick cells following TBEV infection. IDE8 and IRE/CTVM19 cells were infected with TBEV at MOI 0.1, 1.0 and 5.0 and cells were fixed and immunostained at day 2 p.i. The percentage of E protein-positive cells was calculated. The mean of duplicate cultures is shown. **b** TBEV production over a 10-day time course in tick cell lines. IDE8 and IRE/CTVM19 cells were infected with TBEV at MOI 5 and virus was titrated by plaque assay on PS cells. The mean PFU/ml of duplicate cultures is shown. The limit of detection was 56 PFU/ml in a 10^−1^ dilution

Tick cells are able to support infection with a variety of different viruses; as expected, the dynamics of infection vary according to the virus and the cell line [61–63]. To date only two studies have been published on TBEV using cell lines derived from its natural vector *I. ricinus* [34, 35]. To establish the appropriate MOI and time-points for transcriptomic and proteomic analysis, it was first necessary to determine the MOI at which most of the cells would be infected, thereby preventing uninfected cells from masking the transcriptomic and proteomic changes occurring upon TBEV infection. Two cell lines were studied: IRE/CTVM19 derived from *I. ricinus* and IDE8 derived from *I. scapularis*. To determine the appropriate MOI, tick cells were grown on coverslips in 24-well plates and infected with TBEV at MOI 0.1, 1 or 5. Cells were fixed at day 2 p.i., immunostained for TBEV E protein and the percentage of positive cells calculated (Fig. 1a). MOIs of 0.1 and 1 resulted in approximately 40 % of E protein-positive IRE/CTVM19 cells in comparison to 70 % at MOI 5. In IDE8 cells, however, less than 10 % of cells were E protein-positive when infected with MOI 0.1, 25 % with MOI 1 and 55 % with MOI 5. All currently-available tick cell lines are phenotypically and genotypically heterogeneous [41] and relatively uncharacterised; some cell types within the two cell lines used might not support virus infection or the amount of E protein in some infected cells might be below the detection limit of the assay. The observation that not all tick cells are positive for TBEV upon TBEV infection and that the percentage of infected cells varies according to the cell line is consistent with a previous report on TBEV infection in tick cell lines [34]. Since both tick cell lines showed the highest percentage of TBEV-positive cells with MOI 5, the course of infection at this MOI was determined in greater detail.

To measure newly-produced virus within defined time-periods, tick cells were infected with TBEV at MOI 5. The cells were then washed and fresh medium was added at 2 h p.i. for time-points 12, 18 and 24 h, and at 24 h prior to each sampling for the subsequent daily time-points up to day 10 p.i. (Fig. 1b). Supernatants were collected at each time-point and the virus titre measured by plaque assay on PS cells. The pattern of TBEV infection was similar in both cell lines with the highest level of virus production between 2 and 4 days p.i. (Fig. 1b). Interestingly, the maximum titre in the cell line IRE/CTVM19 derived from a known TBEV vector was approximately 2 log_10_ higher than in the IDE8 cell line. The higher level of virus production in the *I. ricinus* cell line compared to the cell line derived from *I. scapularis*, which is not known to be a natural vector of TBEV, confirms previous studies [31, 34]. The lower virus titres in IDE8 cells might be an indicator of reduced vector capacity of *I. scapularis* for this virus, which could be due to cells being less efficiently infectable or having a more rapid and efficient antiviral response.

In order to examine how these two cell lines react to virus infection, two time-points were chosen for transcriptomic and proteomic analysis: one early in infection at day 2 when virus production was increasing, and one later in infection at day 6 when virus production was decreasing.

### Generation of samples for transcriptomic and proteomic analysis

Six replicate tubes per time-point per cell line were either infected with TBEV at MOI 5 or mock-infected. At days 2 and 6 p.i., both RNA and protein were isolated from each tube by dividing the cell suspension in half, thus ensuring that both transcriptomic and proteomic analyses were carried out on samples derived from each tube. Because of the biosafety restrictions on handling TBEV, it was only possible to generate a single set of samples, which yielded sufficient material for a single sequencing run and proteomic analysis for each treatment. Therefore the results will be considered in this context as a baseline for future studies.

Prior to RNA sequencing, RNA samples were tested for the presence of TBEV by qRT-PCR (Additional file 3). Only mock-infected samples negative for TBEV with NS5 RNA levels below the detection limit of the assay, and infected samples positive for TBEV with NS5 levels above 10,000 copies, were included in the subsequent analysis. Furthermore, only high quality samples showing no signs of RNA degradation were used. The soluble proteins extracted from mock-infected and TBEV-infected IDE8 and IRE/CTVM19 cells at days 2 and 6 p.i. were tested for protein quality prior to pooling. Only those RNA samples and protein samples which passed both the RNA and protein quality checks (Additional file 3) were pooled, guaranteeing that pooled RNA samples and protein samples contained RNA and protein, respectively, from the same original samples.

### Illumina sequencing, assembly and differential gene expression analysis

Aliquots of RNA from two or three replicate samples per time-point per cell line (Additional file 3) were pooled and sequenced on the Illumina HiSeq2000 platform. Totals of 29–37 million and 26–44 million raw reads of 100 bp in length were sequenced for IDE8 and IRE/CTVM19 cells, respectively (Table 1). The raw reads from IDE8 were assembled into 44,474 - 44,907 contigs with a mean length of 938 bp and those of IRE/CTVM19 were assembled into 70,067 - 70,842 contigs with a mean length of 1089 bp (Table 1). The difference in contig numbers between the two cell lines is not unexpected since the former were assembled against the sequenced *I. scapularis* genome while the latter were, in the absence of a genome, *de novo* assembled. From the sequencing data for each cell line almost the complete TBEV genome was *de novo* assembled. Of the total number of reads generated, approximately 3.0 % and 2.8 % aligned to TBEV in infected IDE8 cells at days 2 and 6 p.i. respectively, whereas 4.0 % and 7.7 % aligned to TBEV in infected IRE/CTVM19 cells on days 2 and 6 p.i. respectively. The higher level of viral RNA present within IRE/CTVM19 cells compared to IDE8 cells is in agreement with the greater number of infected cells (Fig. 1a) and the higher infectious virus titre (Fig. 1b) in IRE/CTVM19 cultures. The almost completely-assembled virus genome obtained from the two cell lines was identical at each time-point and in each cell line (data not shown). The assembled virus showed 99 % coverage and 99 % similarity to the TBEV Neudoerfl sequence on NCBI (U27495.1). Although TBEV does not have a poly(A) tail, the Neudoerfl strain of TBEV contains a poly(A) sequence within the variable region of the 3’ non-coding region [64] which might explain its presence in the poly(A)-selected RNA pool used for sequencing.

**Table 1:** Sequencing depth, assembly of RNA-Seq data and total number of proteins identified by MS from TBEV-infected and mock-infected (control) IDE8 and IRE/CTVM19 cells on days 2 (2d) and 6 (6d) p.i

Sample	Mean reads per lane	Total no. of contigs assembled	Mean contig length	Total no. of proteins identified by RP-LC-MS/MS
IDE8 control 2d	3.71E + 07	44562	938	907
IDE8 infected 2d	3.25E + 07	44907	937	770
IDE8 control 6d	3.18E + 07	44684	938	824
IDE8 infected 6d	2.96E + 07	44474	939	725
IRE/CTVM19 control 2d	3.03E + 07	70701	1087	835
IRE/CTVM19 infected 2d	2.70E + 07	70067	1092	762
IRE/CTVM19 control 6d	4.44E + 07	70842	1086	1133
IRE/CTVM19 infected 6d	2.61E + 07	70273	1091	1032

Only raw reads that mapped unambiguously to assembled contigs were counted, and it was assumed that the counts for each contig represented the expression level of each transcript. While the majority of reads mapped unambiguously, this approach could lead to an underestimation of transcript expression; however, this would affect both TBEV-infected and mock-infected samples in a similar way. This approach could create problems if there was a true shift of splice isoforms, with one isoform only present in the infected and the other only in the mock-infected samples. It was not possible to determine whether this phenomenon occurred in the present study. The raw count data was used to determine differential gene expression between each of the infected IDE8 and IRE/CTVM19 samples and their respective mock-infected controls using DESeq [52] in R. This allows for calling significance in samples without replicates [65]. It also uses a very conservative estimation of variance, reducing the number of transcripts called as statistically significant. This focus on statistically significantly differentially-expressed transcripts is a stringent filter which may miss some transcripts. In IDE8 cells at days 2 and 6 p.i., totals of 23 and 21 transcripts respectively were statistically significantly differentially expressed with a majority of genes down-regulated on both days (Table 2). In contrast, in IRE/CTVM19 cells totals of 40 and 43 transcripts were statistically significantly differentially expressed on days 2 and 6 p.i. respectively, with the majority of transcripts being up-regulated on both days (Table 2).

**Table 2:** Number of statistically significantly differentially expressed transcripts that were up- or down-regulated upon TBEV infection of IDE8 and IRE/CTVM19 cells on days 2 (2d) and 6 (6d) p.i

Transcript status	IDE8		IRE/CTVM19	
	2d	6d	2d	6d
Up-regulated	8	7	24	43
Down-regulated	15	14	16	0
TOTAL	23	21	40	43

### Protein identification and differential protein representation

Proteins in pooled samples from 2–3 replicates (Additional file 3) were analysed by RP-LC-MS/MS and identified by searching against the arthropod and *Flaviviridae* Uniprot databases. For IDE8 cells, 725–907 proteins were identified in mock-infected and TBEV-infected samples at days 2 and 6 p.i., with slightly fewer proteins being identified at day 6 p.i. than at day 2 p.i. (Table 1 and Additional file 2). For IRE/CTVM19 cells, 762–1133 proteins were identified in mock-infected and TBEV-infected tick cells, with more proteins being identified at day 6 p.i. than at day 2 p.i. (Table 1 and Additional file 2). In both cell lines, slightly higher numbers of proteins were identified in control cells than in TBEV-infected cells, suggesting that TBEV might have an inhibitory effect on protein representation. The higher number of *I. scapularis* protein sequences present in the arthropod database compared to *I. ricinus* sequences did not influence peptide/protein identification. In addition to the arthropod database, MS spectra were used to search against the *Flaviviridae* database. Considering only those peptides with more than one peptide match (FDR <0.01) against the database, only TBEV-infected samples were positive for TBEV and mock-infected cells were negative (Additional file 2). The presence of TBEV proteins was in accordance with detection of TBEV sequences by RNA-seq and confirmed that infected samples, but not mock-infected samples, were infected and that the level of infection was greater in IRE/CTVM19 cells than in IDE8 cells (Additional file 2). Totals of 52 and 24 proteins were differentially represented in IDE8 cells on days 2 and 6 p.i. respectively, while 20 and 109 proteins were differentially represented in IRE/CTVM19 cells on days 2 and 6 p.i. respectively (Table 3). Overall, more proteins were differentially represented in IRE/CTVM19 cells than in IDE8 cells, reflecting the difference observed in gene expression between the two cell lines.

**Table 3:** Number of statistically significantly differentially-represented proteins upon TBEV infection of IDE8 and IRE/CTVM19 cells on days 2 (2d) and 6 (6d) p.i

Protein status	IDE8		IRE/CTVM19	
	2d	6d	2d	6d
Over-represented	20	14	10	24
Under-represented	32	10	10	85
TOTAL	52	24	20	109

### Annotation and ontology of tick cell transcripts and proteins

**Fig. 2:**
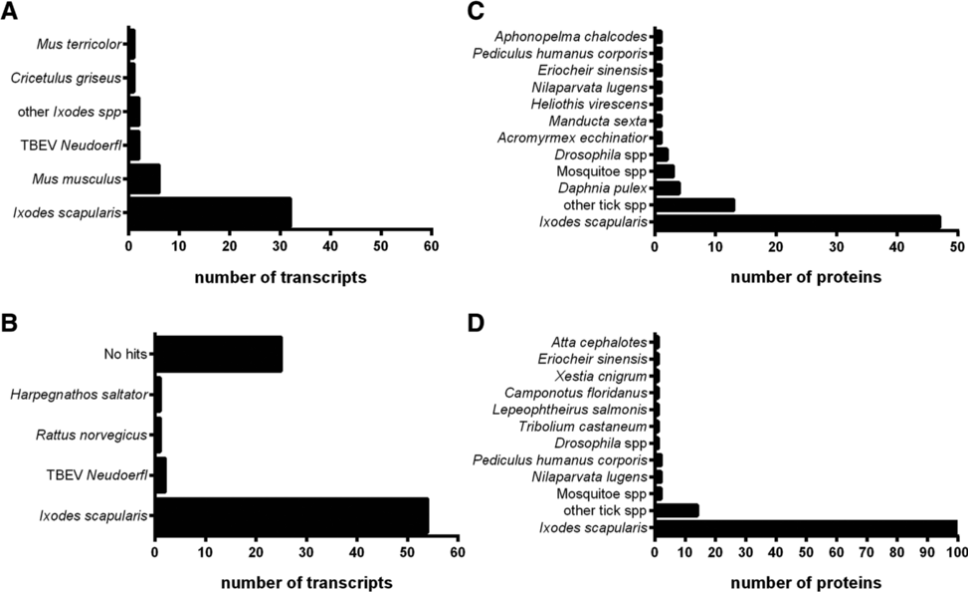
Species distribution of blast hits for differentially-expressed transcripts and differentially-represented proteins in TBEV-infected IDE8 and IRE/CTVM19 cells in the nt and nr database. The numbers of transcripts (**a** and **b**) and proteins (**c** and **d**) with homology to published sequences from *I. scapularis*, other tick species, other arthropod species and vertebrate species are shown for IDE8 (A and C) and IRE/CTVM19 (B and D). Transcript numbers on the x-axes were determined by combining results from both time-points. Species to which the transcripts and proteins showed the highest homology are shown on the y-axes

The majority of blast hits obtained for both transcripts and proteins of IDE8 and IRE/CTVM19 cells corresponded to *I. scapularis* (Fig. 2), which is not surprising since the majority of tick sequences deposited in databases to date were derived from *I. scapularis*, which is the only tick species with a sequenced genome.

In IDE8 cells, all 42 statistically significantly differentially-expressed tick cell transcripts (Fig. 2a) were annotated; 32 were most closely related to transcripts from *I. scapularis,* 2 to transcripts from other *Ixodes* spp, and 8 to transcripts from rodent species. In IRE/CTVM19 cells (Fig. 2b), only 56 of the 81 statistically significantly differentially-expressed tick cell transcripts could be annotated; 54 corresponded to *I. scapularis* and one each to *Rattus norvegicus* and *Harpegnathos saltator*. The other 25 transcripts did not return any blast hits and were excluded from further analysis. This lack of homology has been reported in other tick studies [66–68] and might be attributed to factors such as low sequence and/or assembly quality, lack of homology to the *I. scapularis* genome due to its fragmented state or that these sequences represent novel species-specific transcripts [68].

With respect to the statistically significantly differentially-represented proteins, the majority in both IDE8 (Fig. 2c) and IRE/CTVM19 (Fig. 2d) corresponded to *I. scapularis* followed by other tick species including *Amblyomma* spp., *Hyalomma marginatum rufipes* and *Haemaphysalis qinghaiensis*.

The *I. scapularis* genome is currently not fully annotated, and annotation of transcripts or proteins, including the inference of their functional role, relies in the majority of cases on sequence similarity to evolutionarily quite distant species, including mammals and insects with well-annotated genomes. Thus although homology is observed for a specific transcript or protein, it might have evolved functions within the tick different from those within other species. This comparative approach might therefore be misleading and makes it difficult to infer true biological role by sequence similarity, conserved domains and/or literature search. Currently the only method to identify possible target genes within large datasets of ticks is to infer biological function from other better-annotated organisms or from sequence similarity to other model or non-model organisms.

**Fig. 3:**
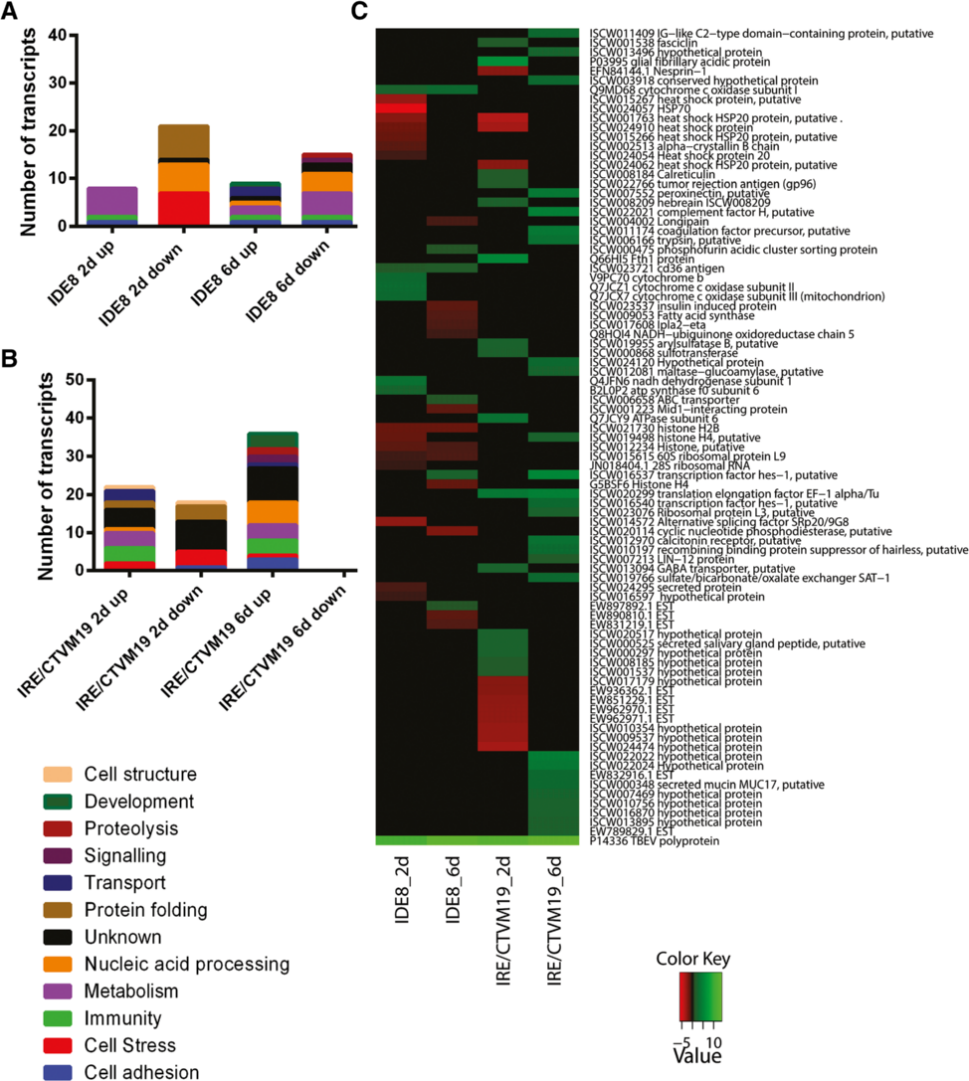
Gene ontology and expression profiles of differentially-expressed transcripts in TBEV-infected IDE8 and IRE/CTVM19 cells. Each individual transcript of IDE8 (**a**) and IRE/CTVM19 (**b**) differentially expressed on days 2 (2d) and 6 (6d) p.i. was assigned to a biological process group according to its regulation status (up or down). Biological process (ontology) groups were assigned using information available on UniProt/Swiss-Prot databases, and were then manually curated according to gene function published in the literature. **c** Differentially-expressed transcripts in IDE8 and IRE/CTVM19 and their levels of differential expression on days 2 (2d) and 6 (6d) p.i. are depicted in the heatmap. Green = up-regulation; red = down-regulation; black = no change. Numbers on the colour key indicate the log_2_-fold change in differential expression

**Fig. 4:**
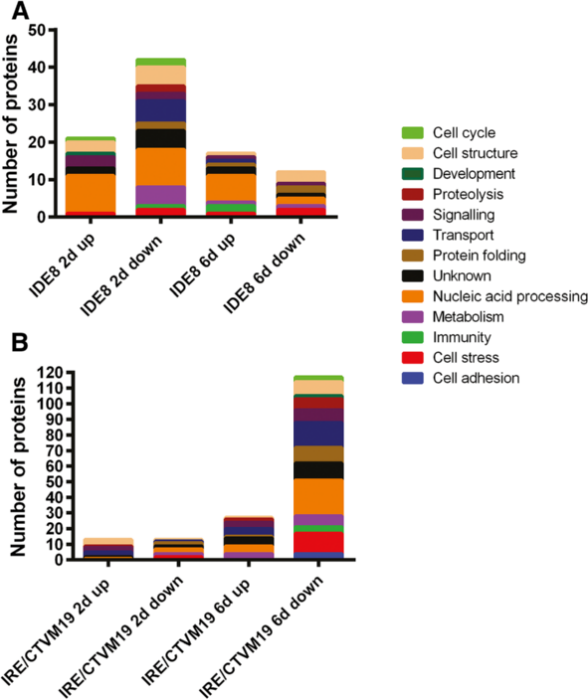
Ontology of differentially-represented proteins in TBEV-infected IDE8 and IRE/CTVM19 cells. Each individual protein of IDE8 (**a**) and IRE/CTVM19 (**b**) differentially represented on days 2 (2d) and 6 (6d) p.i. was assigned to a biological process group according to its regulation status. Ontology groups were assigned using information available on UniProt/Swiss-Prot databases, and were then manually curated according to gene function published in the literature

To allocate annotated differentially-expressed transcripts and differentially-represented proteins to biological process groups, ontology information, including information for conserved domains, was retrieved from the UniProt/Swiss-Prot and Panther databases. Ontology information was manually augmented and/or curated using literature search. Some of the transcripts and proteins were grouped into more than one biological process. In IDE8 the most abundant subcategories within biological processes were nucleic acid processing (23 %), metabolism (23 %) and cell stress (21 %) at the transcript level (Fig. 3a), and nucleic acid processing (30 %), transport (22 %) and cell cycle (20 %) at the protein level (Fig. 4a). In IRE/CTVM19, ignoring those transcripts with no blast hits (30 %), the majority of transcripts were of unknown ontology (26 %) followed by those involved in immunity (10 %), transport (10 %) and cell stress (8 %) (Fig. 3b). At the protein level the majority were classed into nucleic acid processing (25 %), transport (22 %) and cell stress (15 %) (Fig. 4b).

In both cell lines, high levels of virus (Fig. 1b) were associated with up-regulation of transcripts annotated as immunity or metabolism and down-regulation of transcripts annotated as cell stress and protein folding. At the protein level, proteins annotated as cell stress or protein folding were, as with transcripts, amongst those consistently down-regulated. Equivalent biological process groups, as well as specific transcripts and proteins, have been observed to be differentially expressed in studies of mosquitoes and mosquito cells upon virus infection [65, 69, 70]. Although some of these studies showed different directions of representation, this might be attributed to different sampling times, different host and/or virus species and/or *in vivo* versus cell line usage. When comparing the transcript and protein profiles for each cell line at each time-point individually, there is little correlation at the biological process group level (Fig. 3a and b compared to Fig. 4a and b) between transcripts and proteins, apart from those involved in protein folding and cell stress, both of which are generally down-regulated in both TBEV-infected cell lines at both time-points. This lack of correlation between statistically significantly differentially-expressed transcripts and differentially-represented proteins was also observed in studies on tick cell responses to infection with intracellular bacteria [67, 71, 72], and probably reflects the different half-lives of mRNA and proteins and differential regulation of systems at the transcriptional, post-transcriptional, translational or post-translational levels. Novel approaches could increase the correlation between these datasets, for example proteomics informed by transcriptomics [73] which has recently been applied to ticks *in vivo* [51, 74].

**Fig. 5:**
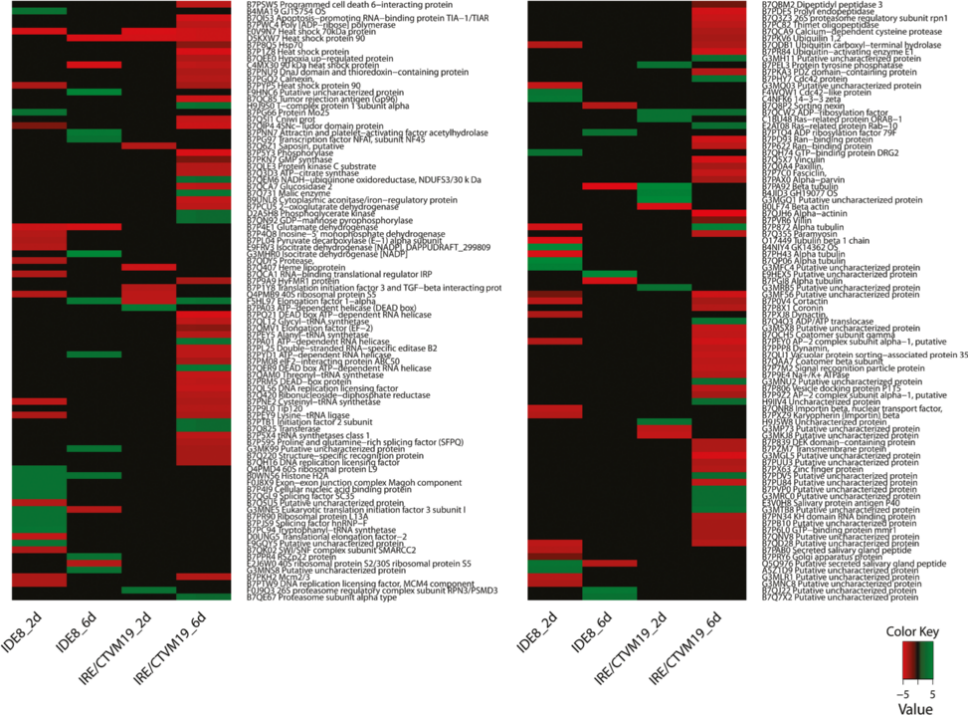
Representation profiles of differentially-represented proteins in TBEV-infected IDE8 and IRE/CTVM19 cells. Differentially-represented proteins in IDE8 and IRE/CTVM19 and their levels of differential representation on days 2 (2d) and 6 (6d) p.i. are depicted in the heatmap. Green = over-represented; red = under-represented; black = no change. Numbers on the colour key indicate the log_2_-fold change in differential representation

Comparing the response to TBEV infection of IDE8 cells with that of IRE/CTVM19 cells, it is apparent that the two cell lines respond differently. Both the actual differentially-expressed transcripts (Fig. 3c) and differentially-represented proteins (Fig. 5), and their expression/representation levels, were different. The differential response at the transcript level might be, at least in part, an artefact resulting from the necessity for using different assembly approaches for the two cell lines, with *de novo* assembly for IRE/CTVM19 and mapping against a reference genome for IDE8; however, a recent study investigating the effect of these two different approaches on differential gene expression found that they usually agree well with each other [75]. Furthermore, the same method of protein identification and statistical analysis was used for both cell lines, and thus the different response is more likely to be due to cell line-specific differences.

To validate the differential gene expression observed during RNA-Seq, twelve transcripts differentially expressed in the transcriptomics data, and/or coding for differentially represented proteins in the proteomics data, were selected for qRT-PCR analysis. Preference was given to those putatively involved in immunity or cell stress, and transcripts/proteins with a range of different expression levels were chosen. Transcripts for the housekeeping genes ribosomal protein L13A and beta actin were used for normalisation since neither was differentially expressed in either of the cell lines at either time-point in the transcriptomic data. Most of the twelve transcripts showed similar fold changes by qRT-PCR and RNA-Seq, or at least confirmed the trend seen in the sequencing data (Additional file 4). The differences observed between the two techniques qRT-PCR and RNA-Seq for complement factor H in both cell lines and coagulation factor in IDE8 cells at day 6 p.i. (Additional file 4, A) might result from splice variants of these transcripts, different RNA processing techniques, primer design, the choice of reference genes and/or the different normalisation methods – normalisation across the whole transcriptome for sequencing depth with RNA-Seq versus normalisation against specific transcripts with qRT-PCR [76]. Similar observations, in which the differential gene expression data by RNA-Seq was, for most transcripts, in good agreement with qRT-PCR data in showing at least a similar trend in differential expression, have also been reported in other transcriptomic studies [77–79].

### Functional role of selected cell stress and immunity genes and proteins in tick cells during LGTV infection

**Fig. 6:**
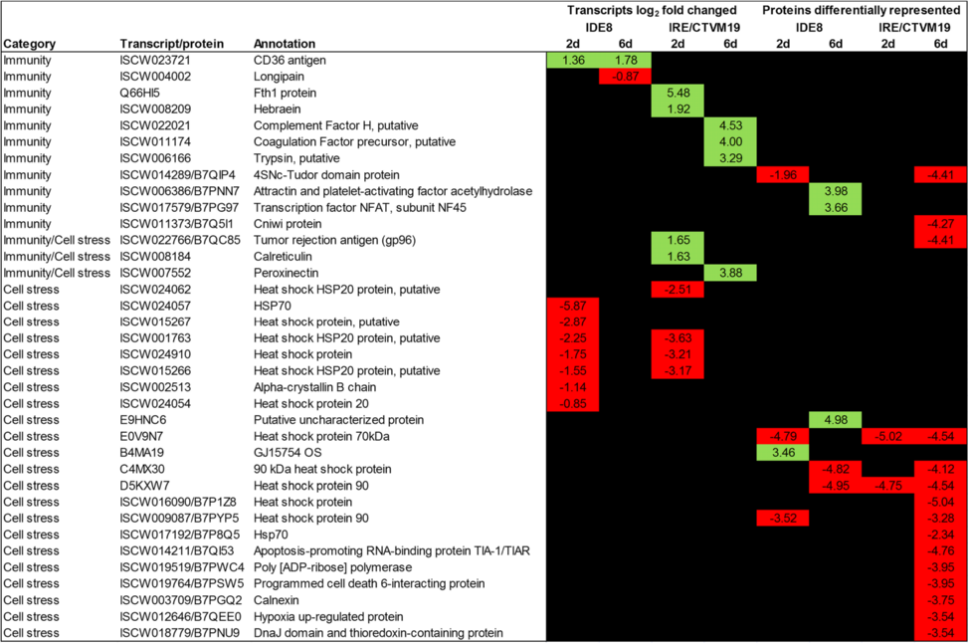
Transcripts and proteins putatively involved in immunity and cell stress in TBEV-infected IDE8 and IRE/CTVM19 cells. Statistically significantly differentially-expressed transcripts and differentially-represented proteins with a possible role in immunity and/or cell stress are listed and their levels of differential regulation in TBEV-infected IDE8 and IRE/CTVM19 cells at days 2 and 6 p.i. are shown. Green = up-regulation/over-representation; red = down-regulation/under-representation; black = no change

The main aim of this study was to identify transcripts and proteins which might have an antiviral role in tick cells. Therefore further analysis was undertaken on differentially-expressed transcripts and differentially-represented proteins with a possible role in innate immunity or cell stress (Fig. 6). For these experiments we used LGTV because it can be used at biosafety level 2, in comparison to TBEV which in many countries has to be handled at a higher level of containment. As it cross-protects against the more pathogenic virus [ 80 ], LGTV is likely to be affected by the same cellular responses as TBEV. To test this hypothesis, IDE8 and IRE/CTVM19 cells were infected with LGTV at MOI 5 or mock-infected with the same culture medium as that used to grow LGTV in Vero cells. At days 2 and 6 p.i., RNA was extracted and transcribed into cDNA. The cDNA was used for qPCR analysis using the same primers that were used for validating differential expression in TBEV-infected cells. Transcript expression in LGTV-infected cells (Additional file 5) revealed a similar trend in differential expression to that of TBEV-infected cells (Additional file 4). However, for some transcripts there was a difference in the level and/or timing of transcript expression. With the exception of complement factor H and coagulation factor, which were both up-regulated in TBEV-infected cells (Additional file 4) and down-regulated in LGTV-infected cells (Additional file 5), the trend in differential expression of all other transcripts was the same in LGTV- and TBEV-infected cells.

Selected transcripts were then silenced using sequence-specific dsRNA in IDE8 and/or IRE/CTVM19 cells, the cells were then infected with LGTV and virus replication and infectious virus production were measured by qRT-PCR and plaque assay respectively. Genes possibly involved in immunity such as those encoding complement factor H or trypsin or those possibly involved in cell stress such as those encoding calreticulin, HSP90, gp96 and HSP70 were silenced in both tick cell lines. Transcripts encoding Argonaute (Ago 30) and Dicer (Dcr 90) were included as positive controls [38], while cells treated with non-specific dsRNA against eGFP were used as baseline controls. Three independent experiments with quadruplicate samples were conducted per cell line; thus 12 samples were analysed in total per cell line. Only those replicates in which silencing was confirmed were included in the statistical analysis.

**Fig. 7:**
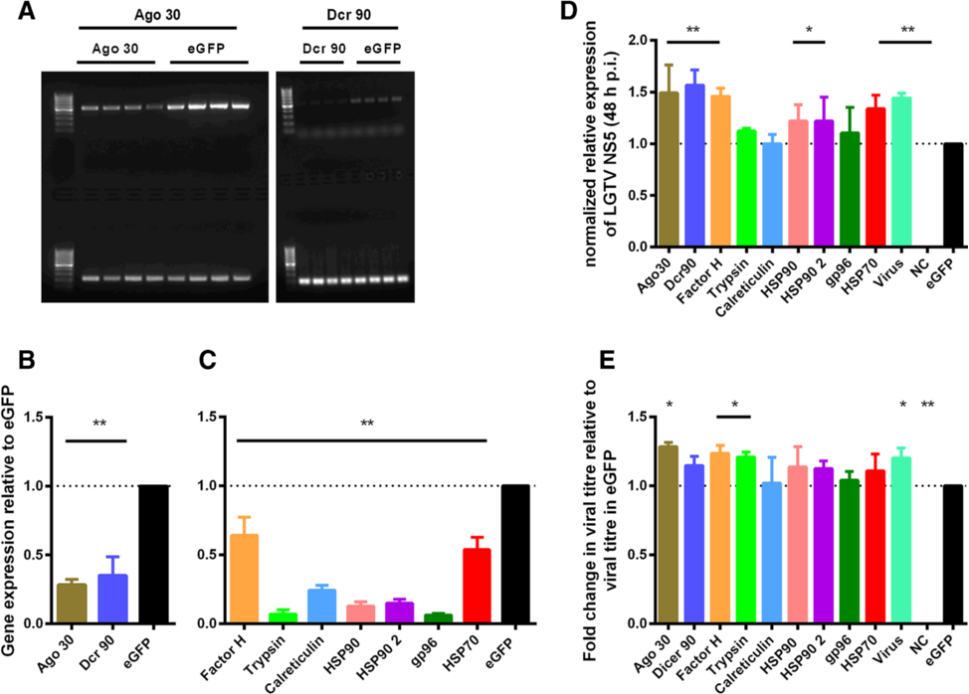
Gene knockdown and the effect on LGTV replication and production in IDE8 cells. IDE8 cells were treated with dsRNA to silence selected transcripts and subsequently infected with LGTV at MOI 0.01. **a** Transcripts coding for Argonaute (Ago 30) and Dicer (Dcr 90) were amplified by RT-PCR using dsT7-Ago 30 or dsT7-Dcr 90 primers and visualised by agarose gel electrophoresis. A representative 1 % agarose gel from one of the three experiments is shown; upper lanes show Ago 30 and Dcr 90 PCR products, lower lanes show beta actin PCR products. **b** Gel-electrophoresis images were used to semi-quantify mRNA knockdown of Ago 30 and Dcr 90 with Image Lab software (BioRad) normalised to beta actin control. **c** Knockdown of mRNA of the genes listed in the x-axis was quantified using qRT-PCR with qRT-PCR primers (Additional file 1). Gene expression was normalised to beta actin and is shown relative to eGFP-dsRNA controls. **d** Viral RNA levels were determined by qRT-PCR using LGTV NS5 primers at 48 h p.i.. The data was normalised to beta actin and is presented for each of the genes listed in the x-axis, and for cells that were not treated with any dsRNA and then infected with LGTV (Virus), as fold change relative to eGFP dsRNA controls. **e** Infectious virus present in the supernatant was titrated by plaque assay at 48 h p.i. and the titres are presented for each of the genes listed in the x-axis, and for cells that were not treated with any dsRNA and then infected with LGTV (Virus), as fold change relative to titres in the eGFP-dsRNA control. The mean with standard error of three independent experiments is shown, including only those replicates in which the knockdown was validated. Statistical significance was calculated using two-way ANOVA Fisher’s LSD test (* *p* < 0.05, ** *p* < 0.001)

**Fig. 8:**
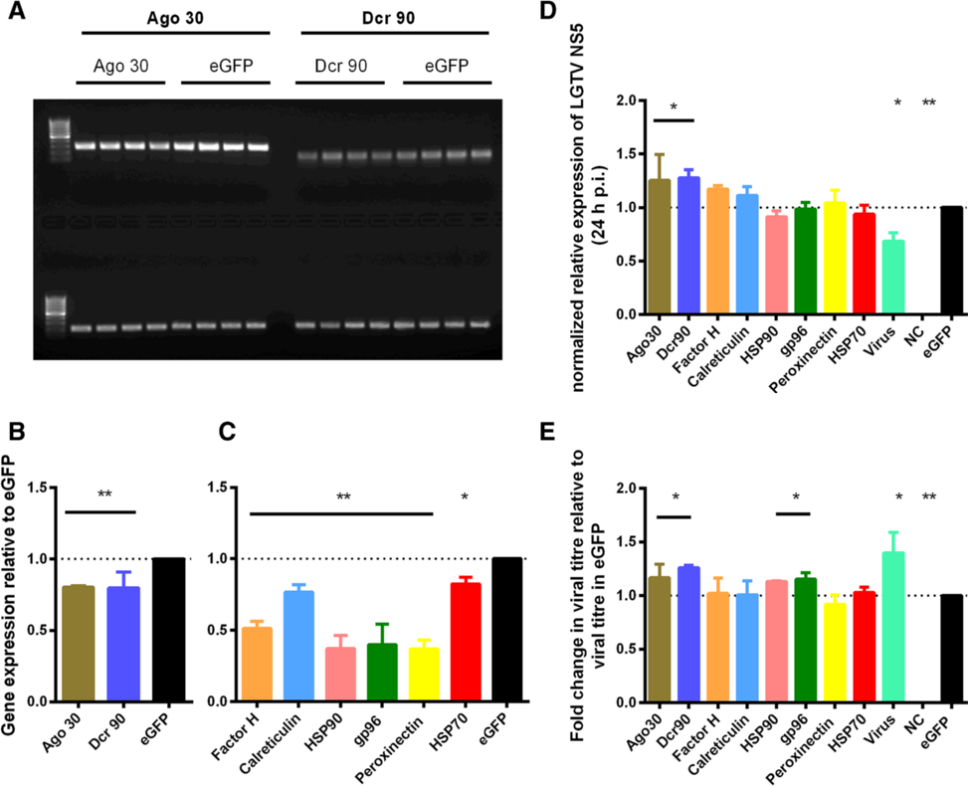
Gene knockdown and the effect on LGTV replication and production in IRE/CTVM19 cells. IRE/CTVM19 cells were treated with dsRNA to silence differentially-expressed transcripts and were subsequently infected with LGTV at an MOI of 0.5. **a** Transcripts coding for Argonaute (Ago 30) and Dicer (Dcr 90) were amplified by RT-PCR using dsT7-Ago 30 or dsT7-Dcr90 primers and visualised by agarose gel electrophoresis. A representative 1 % agarose gel from one of the three experiments is shown; upper lanes show Ago 30 and Dcr 90 PCR products, lower lanes show beta actin PCR products. **b** Gel-electrophoresis images were used to semi-quantify mRNA knockdown of Ago 30 and Dcr 90 with Image Lab software (BioRad) normalised to beta actin control. **c** Knockdown of mRNA was quantified using qRT-PCR. Gene expression was normalised to beta actin and is shown relative to eGFP-dsRNA controls. **d** Viral RNA levels were determined by qRT-PCR using LGTV NS5 primers at 24 h p.i.. The data was normalised to beta actin and is presented for each of the genes listed in the x-axis, and for cells that were not treated with any dsRNA and then infected with LGTV (Virus), as fold changes relative to eGFP dsRNA controls. **e** Infectious virus present in the supernatant was titrated by plaque assay at 24 h p.i. and the titres are presented for each of the genes listed in the x-axis, and for cells that were not treated with any dsRNA and then infected with LGTV (Virus), as fold change relative to titres in the eGFP-dsRNA control. The mean with standard error of three independent experiments is shown, including only those replicates in which the knockdown was validated. Statistical significance was calculated using two-way ANOVA Fisher’s LSD test (* *p* < 0.05, ** *p* < 0.001)

In IDE8 cells, silencing was confirmed in all 12 replicate samples treated with dsRNA against Ago 30, trypsin, HSP90, HSP902 and gp96 with 44-98 % efficiency, 11/12 replicates for calreticulin and HSP70 with 31-85 % efficiency and 9/12 replicates for Dcr 90 and complement factor H with 7-100 % efficiency; silencing efficiencies are shown in Fig. 7a, b and c. Variability in knockdown efficiency and consistency for individual transcripts has also been observed in other studies on tick cells [67] as well as studies on other arthropods [81–83]. Variability could be due to tick cells counteracting the RNAi response by increasing transcription, transcripts being differentially protected from RNases, particular dsRNAs being efficiently degraded before achieving a knockdown or target mRNAs being too transient [81]. In IRE/CTVM19 cells, knockdown of transcripts was generally less efficient and consistent than in IDE8 cells, ranging from 5-85 % silencing efficiency and between 6 and 12 replicates showing silencing, depending on the target transcript, over three independent experiments; silencing efficiencies are shown in Fig. 8a, b and c.

RNAi is probably the most important antiviral response in insects [9]. The importance of the RNAi response in the antiviral defence response in tick cells was recently confirmed by detecting specific viRNAs in tick cells infected with LGTV and observing a proviral effect upon silencing of orthologues of key members of the RNAi pathway (Ago 30 and Dcr 90) [38]. Although Ago 30 and Dcr 90 were not differentially expressed upon TBEV infection in the present study, they were included as positive controls. Both proteins are known to be involved in RNAi [38] and knockdown would be expected to result in an increase in levels of viral RNA as well as infectious virus. In IDE8 cells, silencing of Dcr 90 resulted in a significant increase in LGTV RNA levels and silencing of Ago 30 resulted in a significant increase in levels of both LGTV RNA and infectious virus (Fig. 7d and e). In IRE/CTVM19 cells, significant increases in both LGTV RNA levels (Fig. 8d) and infectious virus (Fig. 8e) were observed following knockdown of both Ago 30 and Dcr 90. This confirms the role of RNAi as an antiviral response in tick cells.

Knockdown of ISCW022021, annotated as complement factor H, resulted in an increase in LGTV RNA and infectious virus in IDE8 cells (Fig. 7) but not in IRE/CTVM19 cells (Fig. 8). Complement Factor H, up-regulated on day 6 p.i. in IRE/CTVM19 cells (Fig. 6), functions in vertebrates as a negative regulator of the alternative pathway of the complement system and as a pattern recognition molecule binding with high efficiency to host-specific molecular signatures, such as heparin and sialic acid, thereby protecting uninfected cells from the complement system [84]. In vertebrates, the complement system is an important innate immune response against different families of viruses [85–87]. However some viruses, such as West Nile virus (WNV), are able to evade the complement system by binding and recruiting Factor H, resulting in decreased complement activation and reduced targeting of virus-infected cells [88]. Although ticks have been shown to have a complement system, with all α2 -macroglobulin family proteins, insect thioester-containing and macroglobulin-related proteins [89, 90], that functions against different types of bacteria [89, 91], nothing is known about the antiviral effect of the tick complement system.

If complement factor H interacts with LGTV in tick cells similarly to WNV in mammalian cells, a decrease in virus replication and/or production would be expected but the opposite was seen in the present study. The increase in viral RNA levels and infectious virus in tick cells upon silencing of complement factor H might have resulted in exhaustion of the complement system and lack of any complement antiviral-activity. The fact that complement factor H was up-regulated in response to virus infection suggests an antiviral role, supported by the observed increase in viral RNA levels and infectious virus upon silencing of complement factor H. The designation of ISCW022021 as complement Factor H by the *Ixodes scapularis* Genome Project [92] might be misleading and, instead of being involved in the complement system, it might be involved in another antiviral innate immune response. Further sequence and functional analyses are required to determine the role of ISCW022021 within the tick cell innate immune response.

Silencing of HSP90 and HSP70 resulted in an increase in LGTV RNA levels in IDE8 cells (Fig. 7). This suggests that HSP90 and HSP70 might be involved in loading of siRNA duplexes into Ago 2, as observed in *Drosophila* [93]; thus knockdown of either protein would lead to an impairment of RNAi, which would result in reduction of degradation of viral RNA, as suggested by the higher viral RNA levels seen in the present study in cultures in which HSP90 and HSP70 were silenced, compared to unsilenced controls. It would be interesting to test whether simultaneous knockdown of both HSP70 and HSP90 would augment the increase in viral RNA levels. Knockdown of trypsin also resulted in a significant increase in LGTV production accompanied by a slight non-significant increase in viral RNA levels. The putative antiviral effect of trypsin might be due to its serine protease activity, since serine proteases are involved in the modulation of several immune signaling pathways [94–96] and, of these, one or more might mediate an antiviral role in tick cells.

In contrast to the results with IDE8 cells, in IRE/CTVM19 cells only silencing of Ago 30 and Dcr 90 resulted in significantly increased virus replication and production (Fig. 8). Silencing of HSP90 and gp96 in IRE/CTVM19 cells resulted in a significant increase in virus production without affecting LGTV RNA levels, suggesting an antiviral role for these proteins at the post-transcriptional level in this cell line. HSP90 and gp96 are both heat-shock proteins which are involved in folding of different client proteins. Inhibition of HSP90 in mammalian cells has been shown to block viral replication [97, 98] and this protein has been proposed to be an important factor in the replication of a wide spectrum of RNA viruses [98]. In the present study in tick cells, however, HSP90 seemed to be involved in the antiviral response with an inhibitory influence on virus RNA levels in IDE8 and at the post-translational level in IRE/CTVM19. The ER-based heat-shock protein gp96 is important for the folding of Toll-like receptors (TLRs) and integrins in mammals and *Drosophila* [99]. The putative antiviral role of gp96 observed in the present study might be due to its capacity for folding TLRs or other client proteins involved in the antiviral response, which upon silencing would cause an increase in virus production.

Silencing of complement factor H, which resulted in increased LGTV replication and production in IDE8 cells, did not show any effect in IRE/CTVM19 cells. This could be due to the less efficient and more variable silencing in the latter cell line, compared to IDE8 cells. Additionally the different responses of the two cell lines to LGTV infection could represent a cell line-specific response towards flavivirus infection; however most of the transcripts tested in silencing experiments were differentially expressed upon TBEV infection in IRE/CTVM19 but not in IDE8. The different responses could indicate a species-specific response since the two cell lines were derived from different tick species, or could be due to the heterogeneity of the cell lines [41] or presence of endogenous viruses. Both IDE8 and IRE/CTVM19 cells are persistently infected with endogenous viruses, St Croix River virus and unidentified reovirus-like particles respectively [100, 101], which could affect the innate immune response towards infection with another virus. The presence of an endogenous virus could either suppress or persistently activate certain immune responses thereby affecting silencing of genes and the effect on virus replication and production. Furthermore, each cell line might have a different timing in the response to virus infection, with IDE8 cells possibly activating a response faster than IRE/CTVM19, which could explain the higher virus titres observed for IRE/CTVM19 in the TBEV growth curve experiment.

Interestingly, LGTV production in samples of both cell lines not treated with dsRNA prior to LGTV infection was significantly higher in comparison to samples treated with control dsRNA against eGFP, suggesting that dsRNA treatment alone triggers an antiviral immune response. This is in contrast to studies on mosquitoes and *Drosophila* in which an antiviral response, possibly RNAi, was shown to be triggered by virus-specific dsRNA but not by non-specific dsRNA [102, 103]. A possible explanation for this difference could be the presence of RNA-dependent RNA polymerase in ticks [21] that could be involved in boosting the non-specific antiviral immune response seen in tick cells against non-specific dsRNA. However, studies in other arthropod systems including sandfly cells [104], shrimp [105] and honey bees [106] also showed that non-specific dsRNA can trigger an antiviral state affecting virus infection. Interestingly, dsRNA encoding eGFP resulted in an increase in Dcr 2 levels in *Bombyx mori* [107], suggesting that Dcr 2 recognises dsRNA as a pathogen-associated molecular pattern, which might result in the expression of Vago, an interferon-like molecule inducing an antiviral state in neighbouring cells, as described in *Drosophila* and mosquitoes [108, 109]. Possible homologues of insect Dicers were recently identified in the *I. scapularis* genome and phylogenetic analysis suggests that Dcr 89 is a possible homologue of insect Dcr 2, whereas Dcr 90 is a possible homologue of Dcr 1 [38]. If Dcr 2 and Vago are also induced in tick cells upon addition of non-specific dsRNA, thereby causing an antiviral state in neighbouring uninfected cells and resulting in reduced virus infection levels in the culture overall as observed in the present study, this could suggest a non-specific antiviral response which recognises dsRNA as foreign in tick cells. Vago has been shown to be present within the *I. scapularis* genome [110]; it would therefore be interesting to investigate whether it has a similar function in ticks as it has in insects.

The observation that, in both tick cell lines, silencing of Ago 30, Dcr 90 and HSP90 resulted in increased LGTV NS5 expression and/or virus production strengthens the hypothesis that these proteins are involved in the antiviral response in tick cells. Additionally, the finding that silencing of complement factor H, trypsin and HSP70 in IDE8 and gp96 in IRE/CTVM19 resulted in a proviral effect is encouraging and warrants further experiments to elucidate their roles in the antiviral response in tick cells.

### Other transcripts and proteins that may be involved in innate immunity

In addition to complement factor H and trypsin, which were grouped into the biological process group innate immunity and were functionally analysed in the previous section, several other transcripts and proteins with a possible role in the innate immune response of tick cells to virus infection were differentially expressed/represented. Those most likely to be involved in innate immunity are discussed below; others are discussed in Additional file 6.

Of the differentially-expressed transcripts in IDE8 cells, two were inferred by GO ontology descriptors to be involved in innate immunity: the class B scavenger receptor CD36 and the cysteine protease longipain (Fig. 6). CD36 is a surface receptor on tick haemocytes that is up-regulated upon bacterial infection in *H. longicornis*, where it is involved in granulocyte-mediated phagocytosis of *Escherichia coli* [111]. CD36 has also been suggested to be involved in the RNAi pathway [112]. RNAi is currently the only antiviral pathway known to be effective in ticks [38]. Up-regulation of CD36 in IDE8 cells upon TBEV infection is therefore intriguing and could be an indicator of up-regulation of the RNAi response. Longipain is present in and on the surface of lysosomes in the midgut of the tick *Haemaphysalis longicornis* and, in addition to playing a role in blood digestion, is involved in dose-dependent killing of *Babesia* parasites [113]. Longipain has a high homology to cathepsin B [113], which in mosquitoes is indirectly involved in defence responses against viruses by triggering apoptosis as observed during dengue virus (DENV) infection [83]. It is not known whether longipain has a similar function in ticks.

In IRE/CTVM19 cells, more transcripts with a potential link to an antiviral response were up-regulated than in IDE8 (Fig. 6). One of these, peroxinectin, up-regulated on day 6 p.i. (Fig. 6), is a cell adhesive peroxidase which is stored in haemocyte granules in crustaceans. Upon an immune stimulus, peroxinectin is released from cells by degranulation and is activated by serine proteases to stimulate cell adhesion, encapsulation, phagocytosis and peroxidase activity [114–117]. In the mud crab, white spot syndrome virus (WSSV) infection results in increased expression of peroxinectin within the first 48 h; this increase is associated with a latent period of WSSV infection, suggesting that peroxinectin is involved in the early defence response against this virus [118]. Furthermore, peroxinectin in crustaceans is associated with the prophenoloxidase (proPO) system, as both require the same activating enzyme, a trypsin-like serine protease [119, 120]. However in contrast to other arthropods, ticks are assumed to lack the proPO system, since no proPO-related gene has so far been identified [15, 18], although controversial reports of the existence of this innate immune response in ticks exist [121–123]. Interestingly, the serine protease trypsin was also up-regulated on day 6 in IRE/CTVM19 cells (Fig. 6).

At the protein level, in IDE8 on day 2 and IRE/CTVM19 on day 6, the 4SNc-Tudor (Tudor-SN) domain protein was under-represented. Tudor-SN is part of the RISC complex in *Drosophila*, *Caenorhabditis elegans* and mammals [124] and is involved in binding and possibly cleaving hyper-edited dsRNAs and miRNAs in *Xenopus laevis* and humans respectively [125, 126]. Tudor-SN is expressed, and suspected to be part of the RISC complex, in ticks [21]; it plays a role in tick feeding and the RNAi pathway but does not appear to be involved in innate responses to TBEV or LGTV [127].

In IRE/CTVM19 at day 6 p.i., Cniwi, a PIWI 1 protein, was under-represented. PIWI proteins are part of the piRNA pathway which was initially thought to protect germline cells from transposable elements in *Drosophila*. However, virus-specific piRNA molecules were found to be expressed for a variety of different viruses in *Drosophila* [128] and mosquitoes [129–132], suggesting a possible antiviral role for the piRNA pathway. In a recent study, the antiviral role of PIWI proteins was confirmed when knockdown of PIWI proteins resulted in an increase in Semliki Forest virus replication and production in mosquito cells [132]. It would be tempting to speculate that this pathway might also be important for the antiviral response against TBEV in tick cells. Under-representation of these two proteins in infected cells would be consistent with suppression of RNAi or perhaps customisation of the RNAi system to virus infection.

### Other transcripts and proteins that may be involved in cell stress

In addition to HSP70, HSP90, gp96 and calreticulin, which were grouped into the biological process group cell stress and were functionally analysed above, several other transcripts and proteins involved in cell stress (Fig. 6) might also have an important role in the response of tick cells to virus infection. Those most likely to be involved in cell stress are discussed below; others are discussed in Additional file 6.

Heat-shock proteins are the most abundant and ubiquitous soluble proteins in all forms of life and are involved in a multitude of housekeeping functions essential for cell survival [133]. Studies on tick cell responses to bacterial infection revealed pathogen- and species-specific differences in the expression of HSPs [71, 134]. HSP20 and the small HSP alpha-crystallin B chain were under-represented in both IDE8 and IRE/CTVM19 cells on day 2 p.i. at the transcript level (Fig. 6). In vertebrate cells the generation of large amounts of viral proteins leads, through the unfolded protein response of the endoplasmic reticulum (ER), to cell stress and an increase in HSPs [135, 136]. However, there is evidence that in vertebrates some HSPs may be controlled to disrupt virus replication.

Other proteins involved in cell stress include the three ER chaperones calreticulin, calnexin and gp96. In mammalian cells, calreticulin, a soluble lectin-like chaperone, is involved in Ca^2+^ homeostasis and is important for the processing and maturation of viral glycoproteins [137, 138]. Knockdown of calreticulin in Vero cells reduces the yield of infectious DENV particles [139]. Similarly, knockdown of calreticulin in *Babesia bigemina*-infected *Rhipicephalus microplus* ticks reduced the level of this protozoan parasite [140]. Calnexin, a membrane-bound chaperone of the ER similar to calreticulin, that was up-regulated at the transcript level in IRE/CTVM19 at day 2 p.i., has been shown to be important for viral glycoprotein processing and maturation [138]. In Vero cells, both calnexin and calreticulin have been shown to be important for the production of infectious DENV particles by interacting with the glycosylated DENV E protein, facilitating proper folding and assembly of DENV proteins [139]. Under-representation of calnexin in IRE/CTVM19 cells at day 6 could be interpreted as an antiviral response selected to curtail virus production.

The tumour-rejection antigen gp96 which was up-regulated in IRE/CTVM19 cells on day 2 p.i. and under-represented at the protein level on day 6 p.i. (Fig. 6), is important in mammalian cells for chaperoning TLRs and integrins. Up-regulation of ER chaperones such as gp96 and calreticulin upon virus infection could be a sign of ER stress, which in mammals can lead to triggering of apoptosis or the unfolded protein response leading to inhibition of translation or apoptosis [141]. There is currently no published information on translational inhibition or regulation of the unfolded protein response in ticks.

### Transcripts and proteins that may be involved in nucleic acid processing

Several transcripts and proteins involved in nucleic acid processing functions, such as replication, transcription, processing of nucleic acid or translation, were differently expressed upon TBEV infection in tick cells. This is not surprising since viruses require the nucleic acid processing machinery of the host to amplify their genome and many viruses perturb these processes in cells or manipulate them for their own advantage. Differential regulation of this group of transcripts and proteins was also observed in several other transcriptomic and proteomic studies of arthropods upon virus infection (e.g. [65, 66, 70, 83, 142]). Many of these transcripts and proteins might be involved in replication and translation of TBEV in tick cells and might be interesting targets for future research to understand virus infections in tick cells and ticks. Histones and elongation factor (EF)-1 alpha were differentially represented at both the transcript and protein levels. Several viral proteins have been shown to target histone proteins and host chromatin to interfere with host gene expression by various mechanisms and for different purposes [143]. The C protein of DENV for example targets core histones during infection to disrupt the host cell genetic machinery in favour of viral replication [144]. EF-1 alpha was up-regulated and shown to be important for virus replication in mammalian and mosquito cells during DENV and WNV infection [145–147]. Interestingly, the eukaryotic translation initiation factor eIF3 was under-represented at the protein level in IDE8 cells at day 6 p.i. and in IRE/CTVM19 cells at day 2 p.i. as observed during WNV infection in Vero cells [148]. This is surprising since eIF3 together with the 40S ribosomal subunit, also under-represented in the present study, have been shown to be important during the initial phase of protein synthesis, and flaviviruses are thought to prevent host cell protein shutoff, at least in mammalian cells [149]. However, a recent study using yellow fever virus (YFV) in mammalian cells found that NS5 interacts with eIF3L, a subunit of eIF3, and that overexpression of this subunit facilitates YFV translation but does not affect global protein synthesis [150]. This suggests that eIF3 is also important in tick cells for replication of flaviviruses but that down-regulation of this initiation factor might have an antiviral effect. Furthermore EF-2, t-RNA synthetasen and several other participants in the translation of RNA were also under-represented in the present study.

Another interesting observation is that in both cell lines at both time-points DEAD-box RNA helicase was up-regulated at the protein level which was also seen at the transcriptional level for DENV in *Aedes aegypti* cells [69]. This is interesting, since Dcr 2, a DExD/H-box helicase, was shown to be capable of sensing viral dsRNA in *Drosophila* leading to the production of possibly antiviral molecules [108].

## Conclusions

This is the first study that combines transcriptomic and proteomic analysis to investigate the response of tick cells to infection with a medically important virus. Despite the limitations imposed on the study by biosafety considerations, the findings represent a valuable baseline for future research. Tick cells responded to TBEV infection by changing the expression and/or representation of cellular genes and proteins involved in a variety of biological processes, including metabolism, transport, protein folding, nucleic acid processing, signaling, cell stress and immunity, revealing a complex response of tick cells to virus infection at both the transcriptome and proteome levels, as observed in other arthropods upon virus infection [65, 66, 69, 70, 83, 142, 148, 151–153]. Some of these transcripts and/or proteins, such as those involved in nucleic acid processing, transport, metabolism, protein folding and cell stress, have also been identified in other species as important host cell factors exploited by viruses to support their life cycle in processes including endocytosis, trafficking, viral RNA transcription and translation and virus maturation. Further analyses of these transcripts/proteins using techniques such as Western blotting, although limited by the lack of specific antibodies to tick proteins, might reveal specific factors required for successful infection, replication and production of viruses in ticks and tick cell lines. The dataset created in the present study represents an important starting point for elucidating the viral life cycle and virus-vector relationships.

Several of the identified transcripts and/or proteins have a possible role in immune-related pathways such as the ubiquitin-proteasome pathway, phagocytosis, the complement system, the piRNA pathway and the unfolded protein response. It is obvious from the present study that RNAi is not the only mechanism involved in the antiviral defence response of ticks and that further research is required to elucidate the cellular mechanisms behind virus infection in tick cell lines and ticks. While some of the changes observed upon virus infection could be a response to any microorganism or to cell stress, others appear to be more specific to virus infection, including a Cniwi protein and the 4SNc Tudor domain protein, which are components of the RNAi pathway. Therefore tick cells seem to be able to respond differently to viral and bacterial infection. This is not surprising since tick cells have been shown to be able to raise a specific response against certain bacterial infections [89, 91].

An unexpected but interesting observation was the down-regulation/under-representation of the heat-shock proteins upon TBEV infection in both tick cell lines. This is surprising since vertebrate and invertebrate HSPs are usually up-regulated upon virus infection and some viruses exploit the presence of HSPs to support virus infection [135, 136]. However, HSPs might also have an antiviral effect due to their implication in loading siRNAs into the RISC complex in *Drosophila*. Thus the down-regulation of HSPs might either be a cellular innate immune response protecting the cell by possibly preventing correct folding of the large number of viral proteins, thereby reducing the production of viral particles, or a response induced by the virus to prevent an efficient RNAi response. Knockdown of the RNAi components Ago 30, Dcr 90, the putative complement component complement factor H, the serine protease trypsin and the HSPs HSP90, HSP70 and gp96, suggests a role for these in the antiviral response of tick cells.

Overall the two cell lines showed a complex expression pattern upon TBEV infection, with differences in expression when compared to each other at both the transcript and protein levels (Fig. 6). These differences could be caused by the necessity of using different methods used to assemble the transcriptomic data, or could represent cell line-specific responses to TBEV infection. One cell line might react more slowly in response to virus infection than the other. Alternatively the response might be species-specific, since the two cell lines are derived from different tick species. Furthermore, both cell lines are heterogeneous, with a range of different cell types present within each culture which could be responding differently to virus infection.

This study enhances the understanding of viral infection of tick cells by identifying transcripts and proteins which may have a role in the innate antiviral defence response of ticks by augmenting or limiting virus production. This preliminary knowledge can be used in future studies to identify important host cell factors required for viral infection, as well as elucidating the innate immune response of tick cells to virus infection.

## Additional files

Additional file 1:
**List of primers used in the quantification of TBEV infection, for validation of differential transcript expression and for preparation of dsRNA for gene knockdown.** (DOCX 22 kb) Additional file 2:
**Protein identification after proteomics analysis.** (XLS 461 kb) Additional file 3:
**TBEV infection levels in mock-infected and TBEV-infected tick cells.** Numbers of copies of TBEV NS5 were determined by qRT-PCR using NS5 primers and the linearised plasmid pJET-NS5 to create a standard curve. Copy numbers were normalised to 1 μg of total RNA. The limit of detection was derived from the number of NS5 copies in the highest dilution which was still detectable with a variance less than one Ct and was normalised to 1 μg of total RNA. (A) IDE8 infected and mock-infected (control) at days 2 (2d) and 6 (6d) p.i. (B) IRE/CTVM19 infected and mock-infected (control) at days 2 and 6 p.i.. Error bars are standard deviations. Samples marked with + passed both RNA and protein quality checks and were used in transcriptomic and proteomic analyses. Additional file 4:
**Validation by qRT-PCR of RNA-Seq data for TBEV-infected IDE8 and IRE/CTVM19 cells.** The fold changes in transcript expression in pooled IDE8 (A) and IRE/CTVM19 (B) samples from RNA-Seq data calculated by DESeq in R at days 2 (2d) and 6 (6d) p.i. were compared to the average fold change obtained by qRT-PCR in 2–3 individual biological replicate samples. The dotted line at fold change 1 represents the cut-off for differential expression. Error bars are standard error of the mean. Additional file 5:
**Differential expression levels of transcripts in LGTV-infected IDE8 and IRE/CTVM19 cells.** The fold changes in transcript expression in IDE8 (A) and IRE/CTVM19 (B) samples infected with LGTV at MOI 5 at days 2 and 6 p.i. were determined by qRT-PCR. The mean of three individual biological replicate samples at days 2 (2d) and 6 (6d) p.i. is depicted. The dotted line at fold change 1 represents the cut-off for differential expression. Error bars are standard error of the mean. Additional file 6:
**Discussion of additional transcripts and proteins that may be involved in innate immunity and cell stress** [154-181].(DOCX 16 kb)
