# Supplementary material for: Ixodes scapularis and Ixodes ricinus tick cell lines respond to infection with tick-borne encephalitis virus: transcriptomic and proteomic analysis
Source: Parasit Vectors. 2015 Nov 18;8:599. doi: 10.1186/s13071-015-1210-x (PMC4652421; doi:10.1186/s13071-015-1210-x)
Supplement: Additional file 1: — List of primers used in the quantification of TBEV infection, for validation of differential transcript expression and for preparation of dsRNA for gene knockdown. (DOCX 22 kb) [file 13071_2015_1210_MOESM1_ESM.docx]

**Additional File 1. List of primers used in the quantification of TBEV infection, for validation of differential transcript expression and for preparation of dsRNA for gene knockdown.**

| **Primer name** | **Sequence 5' to 3' ^a^** | **PCR**  **approach** | **Annealing Temp** |
| --- | --- | --- | --- |
| TBEV NS5 F | GCCGTCACTGGGAACATAGT | qPCR | 55°C |
| TBEV NS5 R | ACACACCTCGTTCCAACTCC | qPCR | 55°C |
| LGTV NS5 F | ACCCAAGACTGCTACGTGTGGAAA | qPCR | 60°C |
| LGTV NS5 R | TGAGGAAGTAAAGGGCCTTGCTGA | qPCR | 60°C |
| beta actin F | AAGGACCTGTACGCCAACAC | qPCR | 58°C |
| beta actin R | ACATCTGCTGGAAGGTGGAC | qPCR | 58°C |
| Ribosomal protein L13A F | \| GTGGGCTGGAAGTACCAGAA \| \| --- \| | qPCR | 58°C |
| Ribosomal protein L13A R | \| CTAGCTGAACCTTGGCTTCG \| \| --- \| | qPCR | 58°C |
| Complement Factor H F | ACATCCTTTGGTGCTGGAAC | qPCR | 58°C |
| Complement Factor H R | CACAACGCTGTCCTCAAAGA | qPCR | 58°C |
| Coagulation Factor F | TTACGATGAAGACCCGAACC | qPCR | 58°C |
| Coagulation Factor R | AGATGGACTTCGACCCTCCT | qPCR | 58°C |
| HSP90 F | AGGACGAGCTCCACAACATC | qPCR | 58°C |
| HSP90 R | CGGACGAACACCTCTTTCTC | qPCR | 58°C |
| gp96 F | GCACAAGTTGCTGAAGGTGA | qPCR | 58°C |
| gp96 R | CGGTTGGTAGTGTCCTCGAT | qPCR | 58°C |
| Peroxinectin F | TCTGCGACAACTCAAACCTG | qPCR | 58°C |
| Peroxinectin R | GTAGTGGCCCTACGTCCAGA | qPCR | 58°C |
| 4SCN-Tudor IRE/CTVM19 F | CTGTCTGGGGACACGGTAGT | qPCR | 58°C |
| 4SCN-Tudor IRE/CTVM19 R | TGGTCTCACTGATGGTCTCG | qPCR | 58°C |
| 4SCN-Tudor IDE8 F | GGCCAAGTACTTCACGGAGA | qPCR | 58°C |
| 4SCN-Tudor IDE8 R | GAACCTCCAGTGACCGTTGT | qPCR | 58°C |
| Calreticulin IRE/CTVM19 F | CCCAAGGTGTACCTCAAGGA | qPCR | 58°C |
| Calreticulin IRE/CTVM19 R | GTAAAAGCGGGCATCTTCAG | qPCR | 58°C |
| Calreticulin IDE8 F | TGAAGCACGAGCAGAACATC | qPCR | 58°C |
| Calreticulin IDE8 R | GCAGGTTCTTGCCCTTGTAG | qPCR | 58°C |
| Trypsin IRE/CTVM19 F | GACACCTACGCCAACAACCT | qPCR | 58°C |
| Trypsin IRE/CTVM19 R | GTGTCGTAGCGGTTGACCTT | qPCR | 58°C |
| Trypsin IDE8 F | CCTGAGATCCTCCTGGTTCA | qPCR | 58°C |
| Trypsin IDE8 R | AGGTTGTTGGCGTAGGTGTC | qPCR | 58°C |
| HSP70 IRE/CTVM19 F | GCCAAGATGAAGGAAACTGC | qPCR | 58°C |
| HSP70 IRE/CTVM19 R | ACATTGAGACCGGCGATAAC | qPCR | 58°C |
| HSP70 IDE8 F | GCTCAGTCCACTTCCTCGAC | qPCR | 58°C |
| HSP70 IDE8 R | ACTTTGTCCTGGATGGATGC | qPCR | 58°C |
| Cniwi F | AGAAGGTGGTGCATGGAAAC | qPCR | 58°C |
| Cniwi R | CACATGACCGTCCATGAGTC | qPCR | 58°C |
| CD36 F | CACGGAGGAGTTCGAGTTCT | qPCR | 58°C |
| CD36 R | CAGCCGAACTTAGTCGAAGG | qPCR | 58°C |
| α-crystallin B F | GCTTCTACATCCAGCCCAAA | qPCR | 58°C |
| α-crystallin B R | TCCGACTTCTCTTCGTGCTT | qPCR | 58°C |
| dsT7-eGFP F | taatacgactcactatagggATGGTGAGCAAGGGCGAGGAGCTGTTC | PCR | 60°C |
| dsT7-eGFP R | taatacgactcactatagggCTGGGTGCTCAGGTAGTGGTTGTCGGGC | PCR | 60°C |
| dsT7-Dcr 90 F | taatacgactcactatagggATCCTCAAGGAGTACAAGCC | PCR | 60°C |
| dsT7-Dcr 90 R | taata cgactcactatagggACAGAGCATTAGGGTCGTC | PCR | 60°C |
| dsT7-Ago 30 F | taatacgactcactatagggACATACGAGCACTGACGG | PCR | 60°C |
| dsT7-Ago 30 R | taatacgactcactatagggTGGTGCAACATTTTATCGA | PCR | 60°C |
| dsT7-Calreticulin F | taatacgactcactatagggACAAGGGCAAGAACCTGCT | PCR | 60°C |
| dsT7-Calreticulin R | taatacgactcactatagggAAGATTGTGCCGGACTTGAC | PCR | 60°C |
| dsT7-gp96-1 F | taatacgactcactatagggCGGACTATGTGACACGGATG | PCR | 60°C |
| dsT7-gp96-1 R | taatacgactcactatagggCTCATGGTGTCCTGGGACTT | PCR | 60°C |
| dsT7-trypsin F | taatacgactcactatagggGACACCTACGCCAACAACCT | PCR | 60°C |
| dsT7-trypsin R | taatacgactcactatagggATGGTCACGTCCTCCTTGAG | PCR | 60°C |
| dsT7-HSP90-1 F | taatacgactcactatagggCTGCCCACAAAGTTGCTGTA | PCR | 60°C |
| dsT7-HSP90-1 R | taatacgactcactatagggCTCCGAGAAAGAGGTGTTCG | PCR | 60°C |
| dsT7-HSP90-2 F | taatacgactcactatagggCGAGAAGGAGGAGATTGCAC | PCR | 60°C |
| dsT7-HSP90-2 R | taatacgactcactatagggGGATGACTCTGGAGCCTCTG | PCR | 60°C |
| dsT7-Peroxinectin F | taatacgactcactatagggCATCGTGCAGTCTCTTGGAA | PCR | 60°C |
| dsT7-Peroxinectin R | taatacgactcactatagggAGAAGTGATCGTTGGCATCC | PCR | 60°C |
| dsT7-Complement Factor H IRE/CTVM19 F | taatacgactcactatagggTCCCCTTCATCTGTCAGGTC | PCR | 60°C |
| dsT7-Complement Factor H IRE/CTVM19 R | taatacgactcactatagggCGAAGCTGGAACGGAAGTAG | PCR | 60°C |
| dsT7-Complement Factor H IDE8 F | taatacgactcactatagggACAAATCGGCCGACACTTAC | PCR | 60°C |
| dsT7-Complement Factor H IDE8 R | taatacgactcactatagggGATGGTGTGCACGGTATGAG | PCR | 60°C |
| dsT7-HSP70 IDE8 F | taatacgactcactatagggTCTCGAACGAACAGGAGAGC | PCR | 60°C |
| dsT7-HSP70 IDE8 R | taatacgactcactatagggACATTGAGACCGGCGATAAC | PCR | 60°C |
| dsT7-HSP70 IRE/CTVM19 F | taatacgactcactatagggGCTCAGTCCACTTCCTCGAC | PCR | 60°C |
| dsT7-HSP70 IRE/CTVM19 R | taatacgactcactatagggTGGGAACCTTCAACTTGACC | PCR | 60°C |

F: Forward; R: Reverse

^a^ Lower case and upper case denote sequences of T7 and primer, respectively.
