# Supplementary figures and images for: Ixodes scapularis and Ixodes ricinus tick cell lines respond to infection with tick-borne encephalitis virus: transcriptomic and proteomic analysis
Source: Parasit Vectors. 2015 Nov 18;8:599. doi: 10.1186/s13071-015-1210-x (PMC4652421; doi:10.1186/s13071-015-1210-x)

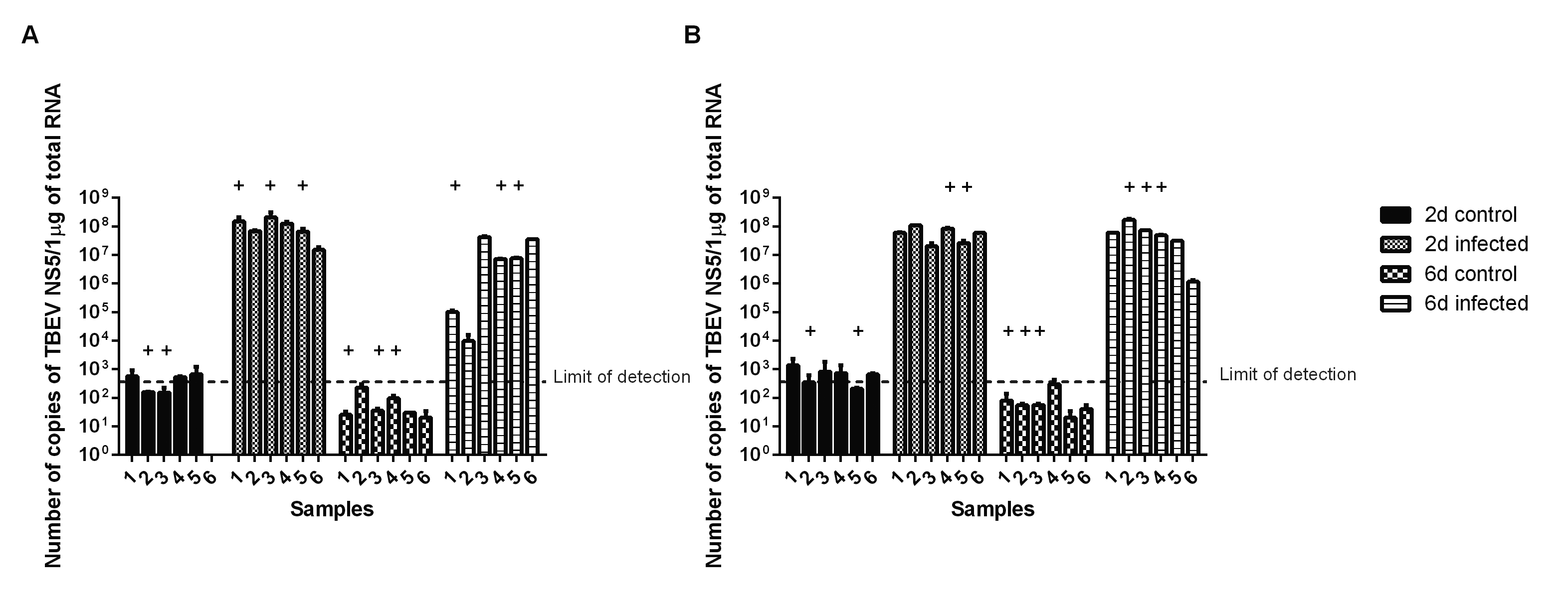

Supplement: Additional file 3: — TBEV infection levels in mock-infected and TBEV-infected tick cells. Numbers of copies of TBEV NS5 were determined by qRT-PCR using NS5 primers and the linearised plasmid pJET-NS5 to create a standard curve. Copy numbers were normalised to 1 μg of total RNA. The limit of detection was derived from the number of NS5 copies in the highest dilution which was still detectable with a variance less than one Ct and was normalised to 1 μg of total RNA. (A) IDE8 infected and mock-infected (control) at days 2 (2d) and 6 (6d) p.i. (B) IRE/CTVM19 infected and mock-infected (control) at days 2 and 6 p.i.. Error bars are standard deviations. Samples marked with + passed both RNA and protein quality checks and were used in transcriptomic and proteomic analyses. [file 13071_2015_1210_MOESM3_ESM.tif]

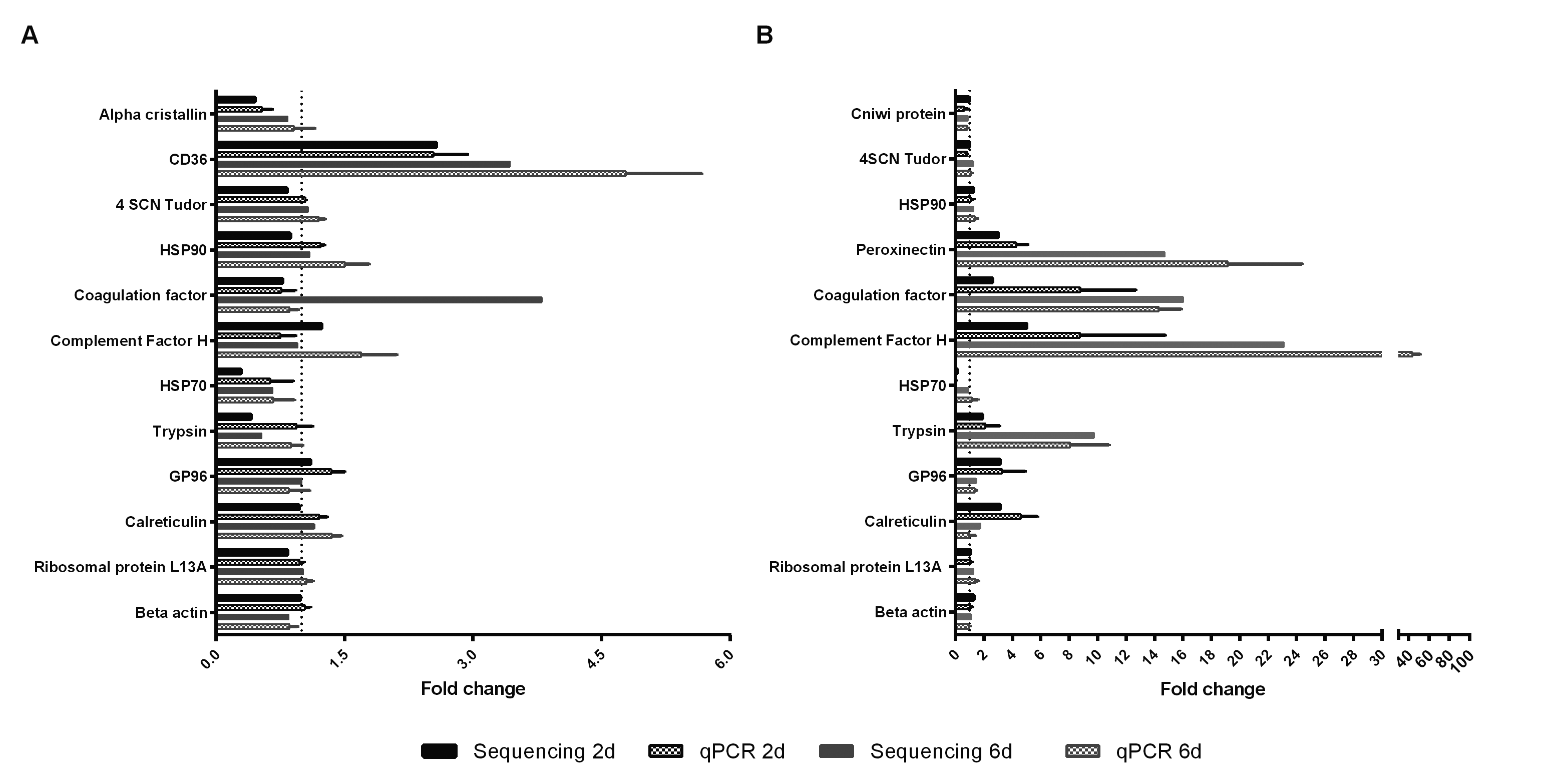

Supplement: Additional file 4: — Validation by qRT-PCR of RNA-Seq data for TBEV-infected IDE8 and IRE/CTVM19 cells. The fold changes in transcript expression in pooled IDE8 (A) and IRE/CTVM19 (B) samples from RNA-Seq data calculated by DESeq in R at days 2 (2d) and 6 (6d) p.i. were compared to the average fold change obtained by qRT-PCR in 2–3 individual biological replicate samples. The dotted line at fold change 1 represents the cut-off for differential expression. Error bars are standard error of the mean. [file 13071_2015_1210_MOESM4_ESM.tif]

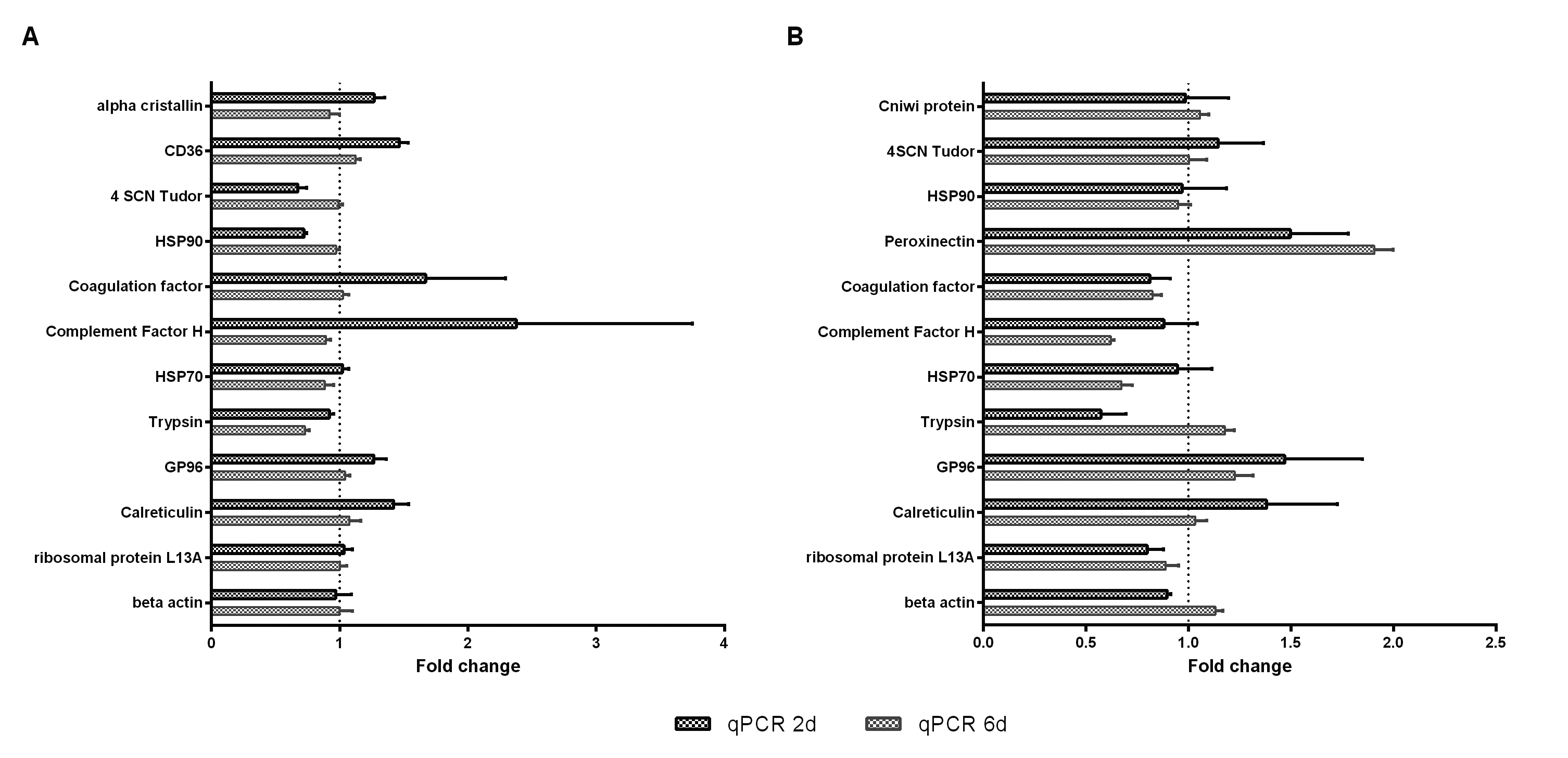

Supplement: Additional file 5: — Differential expression levels of transcripts in LGTV-infected IDE8 and IRE/CTVM19 cells. The fold changes in transcript expression in IDE8 (A) and IRE/CTVM19 (B) samples infected with LGTV at MOI 5 at days 2 and 6 p.i. were determined by qRT-PCR. The mean of three individual biological replicate samples at days 2 (2d) and 6 (6d) p.i. is depicted. The dotted line at fold change 1 represents the cut-off for differential expression. Error bars are standard error of the mean. [file 13071_2015_1210_MOESM5_ESM.tif]
