# Supplementary material for: Ixodes scapularis and Ixodes ricinus tick cell lines respond to infection with tick-borne encephalitis virus: transcriptomic and proteomic analysis
Source: Parasit Vectors. 2015 Nov 18;8:599. doi: 10.1186/s13071-015-1210-x (PMC4652421; doi:10.1186/s13071-015-1210-x)
Supplement: Additional file 6: — Discussion of additional transcripts and proteins that may be involved in innate immunity and cell stress [154-181].(DOCX 16 kb) [file 13071_2015_1210_MOESM6_ESM.docx]

**Additional File 6. Discussion of additional transcripts and proteins that may be involved in innate immunity and cell stress.**

**Transcripts and proteins that may be involved in innate immunity**

To date there is no convincing evidence of the presence of a coagulation cascade in ticks, and although a homologue of Factor C, which triggers the cascade in crustaceans upon bacterial infection, was identified, no homologues of horseshoe crab coagulogen or crustacean clotting protein have been found in ticks [15,18]. Up-regulation in the present study of a putative coagulation factor precursor, which showed 46% identity to horseshoe crab coagulation Factor B that is involved in the horseshoe crab hemolymph clotting cascade, suggests that tick hemolymph clotting, if it exists, could be up-regulated in response to infection.

Two transcripts, hebraein and ferritin, known to be involved in antimicrobial defences, were up-regulated in IRE/CTVM19 on day 2 p.i.. Hebraein, first identified in *Amblyomma hebraeum*, is a histidine- and cysteine-rich antimicrobial peptide similar to microplusin of *Rhipicephalus microplus* [154] that exhibits a bacteriostatic effect by sequestering copper which is required for bacterial respiration [155]. Ferritin, with highest sequence similarity to rat ferritin heavy chain 1, has been previously shown to be up-regulated upon bacterial infection of ticks, suggesting an antimicrobial role by sequestration of iron preventing bacterial multiplication [156–158].

In IDE8 on day 6 p.i., the transcription factor NFAT, subunit NF45 and the platelet-activating factor acetylhydrolase (PAF-AH) were overrepresented. NFAT belongs to a family of transcription factors which are present in cells of the adaptive and innate immune systems in vertebrates, where, upon activation, they induce gene expression regulating several innate and adaptive immune responses [159–162]. In invertebrates, only homologues of vertebrate NFAT5 have been identified [163,164]; these are activated upon osmotic stress [163] or LPS stimulation [164], the latter suggesting a role in LPS-stimulated immunity in invertebrates. PAF-AH, a Ca^2+^-dependent phospholipase A2 involved in inactivation of platelet-activating factor [165,166] and in regulating activation of host cells central to inflammation and hemostasis in vertebrate immunity [167], was identified in the saliva of cat fleas [168]. Furthermore, there is evidence that an increase in PAF-AH in the hemolymph of the insect *Rhodnius prolixus* upon pathogen infection interferes with hemocyte-mediated phagocytosis and hemocyte microaggregation [169,170].

## Transcripts and proteins that may be involved in cell stress

A protein involved in the cell stress response, GJ15754 which exhibits peroxiredoxin activity in *Drosophila* according to electronic annotation with InterPro, was up-regulated on day 2 p.i in IDE8. Peroxiredoxins are antioxidant enzymes protecting cells from oxidative damage by removing reactive oxygen species. Up-regulation of peroxiredoxins was observed in proteomic studies of *Babesia*-infected *R. microplus* ticks [171] and of *Aedes albopictus* mosquitoes and the mosquito cell line C6/36 infected with DENV [70].

Other proteins underrepresented on day 6 p.i. in IRE/CTVM19 that might be involved in the cell stress response included hypoxia up-regulated protein (referred to as GRP170), a DNAJ domain- and thioredoxin-containing protein, poly (ADP-ribose) polymerase (PARP), programmed cell death 6 interacting protein (referred to as Alix/AIP2) and the apoptosis-promoting RNA-binding protein TIA-1/TIAR. The DNAJ domain- and thioredoxin-containing protein was, according to its conserved domains, allocated to both of the biological process groups cell stress and protein folding, but its exact function could not be inferred by literature search. GRP170, a large HSP70 protein present within the ER, functions as a chaperone of unfolded proteins [172]. Nothing is known about the role of these two proteins during virus infection of arthropods. The nuclear enzyme PARP is involved in DNA damage surveillance and promotion of cell death [173]. Alix/AIP2 was found to be involved in promoting apoptosis and endolysosomal trafficking [174]; in mammalian cells infected with vesicular stomatitis virus (VSV), Alix was suggested to control the release of VSV nucleocapsid from late endosomes into the cytoplasm [175], whereas in shrimp Alix does not seem to play a role in viral-induced cell death upon WSSV infection [176]. TIA-1 and TIAR are multifunctional, nucleocytoplasmic shuttling proteins and are involved in apoptosis and the formation of stress granules in mammals and may regulate the lifecycle of mRNAs [177,178]. Many viruses interfere with stress granules by either blocking their formation or co-opting them in order to support virus replication [179,180]. WNV, for example, interacts with TIA-1/TIAR in mammalian cells [181] and blocks stress granule formation [149], which supports virus replication, since TIA-1/TIAR knock-out mice show reduced WNV replication [181].
